# Non‐Random Distribution of EMS‐Induced Mutations Reveals Preference for Open Chromatin and Expressed Genes in Rice

**DOI:** 10.1002/advs.202510034

**Published:** 2025-08-13

**Authors:** Xue‐Feng Yao, Yanhong Liu, Zhiyong Li, Guo‐Qiang Jiang, Wensheng Wang, Hong Lu, Huihui Li, Zefu Lu, Chun‐Ming Liu

**Affiliations:** ^1^ Key Laboratory of Plant Molecular Physiology State Key Laboratory of Forage Breeding‐by‐Design and Utilization Institute of Botany, Chinese Academy of Sciences Beijing 100093 China; ^2^ State Key Laboratory of Crop Gene Resources and Breeding Institute of Crop Sciences Chinese Academy of Agricultural Sciences Beijing 100081 China; ^3^ China Golden Marker Co., LTD Beijing 102206 China; ^4^ School of Advanced Agricultural Sciences Peking University Beijing 100091 China; ^5^ Present address: Maize Research Institute Beijing Academy of Agriculture and Forestry Sciences Beijing 100097 China; ^6^ Present address: Institute of Genetics and Developmental Biology Chinese Academy of Sciences Beijing 100101 China; ^7^ Present address: Agricultural Genomics Institute at Shenzhen Chinese Academy of Agricultural Sciences Shenzhen 518000 China

**Keywords:** chromatin accessibility, EMS mutagenesis, natural variations

## Abstract

Mutagenesis is an effective strategy to generate genetic resources for functional genomics and crop improvement. It is widely accepted that chemical‐induced mutations are randomly distributed across the genome. However, the randomness of such mutations remains a matter of scientific debate due to a lack of systematic investigation. Although the basic features of mutations induced by different mutagens are known, it remains unresolved whether these mutations correlate with the chromatin state and gene expression. In this study, genomes of the wild‐type and 4,619 mutagenized M3 lines derived from treatment with two concentrations of ethyl methane sulfonate (EMS) are sequenced, identifying over 7 million single‐nucleotide mutations (SNP^m^s) that covered over 97% of genes in the rice genome. Comprehensive analyses integrated with genomic, epigenomic, and expression data revealed that SNP^m^s are more frequently located in open chromatin regions and actively expressed genes. Within expressed genes, heterozygous SNP^m^s are enriched both upstream and downstream of transcription start sites (TSSs), whereas homozygous SNP^m^s are enriched significantly in the upstream regions. These findings demonstrate that EMS‐induced mutations are non‐randomly distributed across the rice genome, with a preference for functionally important genomic regions.

## Graphical abstract

Mutagenesis is an effective method for generating genetic resources with a long history in breeding; however, the randomness of chemical‐induced mutations has not been systematically investigated. In this study, over 7 million SNP^m^s are identified in an EMS‐induced population, revealing that these mutations are non‐randomly distributed and preferentially occur in functionally important genomic regions.

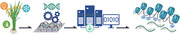

## 1 Introduction

Mutagenesis is a powerful tool for generating genetic variation in support of functional genomics and crop improvement.^[^ ^1^, ^2^ ^]^ Among chemical mutagens, EMS is widely used and primarily induces G/C‐to‐A/T transitions by alkylating guanine residues during DNA replication and repair.^[^ ^3^ ^]^ Traditionally, EMS‐induced mutations, as well as chemical mutagenesis, have been considered to occur randomly across the genome.^[^ ^3^, ^4^, ^5^ ^]^ However, findings from Targeting Induced Local Lesions In Genomes (TILLING) and small‐scale sequencing studies^[^ ^6^, ^7^ ^]^ suggest the presence of mutation biases at specific amino acid sites or genomic regions, leaving the randomness of chemically induced mutations an open question.

Recent studies of spontaneous mutations have suggested that chromatin accessibility and epigenomic features may influence mutation rates.^[^ ^8^ ^]^ While such findings provide intriguing insights, the low frequency of spontaneous mutations poses challenges for comprehensive analysis.^[^ ^9^ ^]^ By contrast, EMS‐induced mutagenesis introduces a high density of mutations across the genome, enabling systematic and high‐resolution investigation of mutation distribution patterns. Given that chromatin accessibility shapes transcriptional regulation and defines functional genomic regions, it remains unclear whether chemical mutagens such as EMS exhibit similar biases.

To address this, we generated a large‐scale mutagenized rice population (Rice4619), consisting of 4619 M2 lines derived from the *japonica* cultivar Zhonghua11. Whole‐genome sequencing of their M3 seeds identified ≈7.4 million SNP^m^s spanning nearly the entire gene space. By integrating epigenomic and transcriptomic datasets, we found that EMS‐induced mutations were significantly enriched in accessible chromatin and actively expressed genes, with clear positional preference near TSSs. These findings demonstrate that EMS‐induced mutations are not randomly distributed but preferentially occur in functional genomic regions, challenging conventional assumptions and providing a framework for more efficient mutagenesis‐based breeding strategies.

## 2 Results

### 2.1 General Features of SNP^m^s in Rice4619

**Figure 1:**
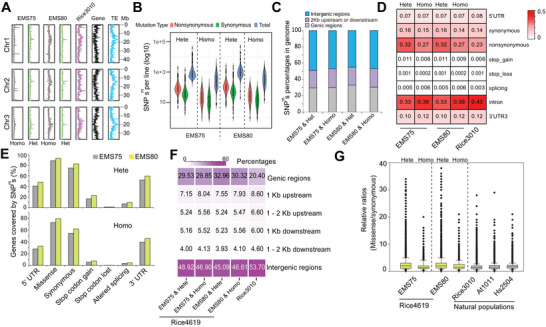
Features of SNP^m^s in Rice4619. A) Line plots showing the distributions of homozygous and heterozygous SNP^m^s in Rice4619, natural SNPs in Rice3010, gene densities, and transposable element (TE) across chromosomes 1, 2, and 3 of the rice genome, calculated in 100 Kb windows. B) Distributions of homozygous and heterozygous SNP^m^s and missense mutations per line in two EMS‐induced populations (EMS75 and EMS80) of Rice4619. C) Frequencies of homozygous and heterozygous SNP^m^s located in different genomic regions. D) Frequencies of various types of mutations and genetic variations within genic regions. E) Proportions of genes affected by different types of mutations. F) Heatmap illustrating the percentages of SNP^m^s/SNPs located in genic and different intergenic regions in Rice4619 and Rice3010. Notably, SNP^m^s in Rice4619 show higher percentages in genic regions and lower percentages in intergenic regions compared with SNPs in Rice3010. G) Boxplot comparing the ratios of missense to synonymous SNP^m^s in Rice4619 with those of natural SNPs in Rice3010, 1001 *Arabidopsis* accessions (At1001),^[^ ^13^ ^]^ and 2504 human samples (Hs2504).^[^ ^14^ ^]^ Homo, homozygous SNP^m^s; Hete, heterozygous SNP^m^s.

Mutagenesis was performed by treating rice ZH11 seeds with 75 and 80 mm EMS (named EMS75 and EMS80, respectively). All M1 plants that produced sufficient seeds were advanced to the M2 generation, with each line sown to generate subpopulations of EMS75 and EMS80 (Figure S1 and Table S1, Supporting Information). Some detectable phenotypes in M2 lines were documented in the field (Table S2, Supporting Information). M3 seeds were harvested, and genomic DNAs were extracted from them to generate a population Rice4619 with 4619 M2 lines: 2209 from EMS75 and 2410 from EMS80 (Table S3, Supporting Information). DNAs from these lines were sequenced individually, with an average sequencing depth of 11.8× and >90% of bases having a Phred quality score ≥ Q30 (Table S3, Supporting Information), and aligned to the ZH11 reference genome.^[^ ^10^ ^]^ Genomic variations present in more than ten lines, as well as insertions or deletions (InDels) larger than 10 bp, were excluded. In total, 7407200 SNP^m^s and 1291355 InDels were identified (**Figure** **1**A; Figure S2 and Table S4, Supporting Information). InDels were excluded from subsequent analysis due to the unclear mechanisms underlying their formation and because they are not the primary mutation type associated with EMS‐induced mutagenesis. Deleterious or lethal mutations can be maintained in a heterozygous state but not a homozygous state;^[^ ^11^ ^]^ therefore, we categorized SNP^m^s into heterozygous and homozygous types to analyze their distributions. To estimate the efficacy of mutagenesis in Rice4619, the average numbers of SNP^m^s per line were calculated to be 1496 for EMS75 and 1702 for EMS80, equating to one SNP^m^ per 252 Kb for EMS75 and per 221 Kb for EMS80 (Figure 1B; Table S4, Supporting Information), and a fraction of these SNP^m^s represented missense mutations that resulted in amino acid changes (Figure 1B). These densities are comparable to those reported for the Nipponbare background TILLING population,^[^ ^12^ ^]^ which are 1/294 Kb (1.5% EMS) and 1/265 Kb (Az‐MNU). In summary, an average of 20 SNP^m^s was identified per 1 Kb of the rice genome across the entire Rice4619 population.

To evaluate the sequencing accuracy, 55 SNP^m^s were selected for re‐sequencing after PCR amplifications, and all of them were validated (Table S5, Supporting Information). A diversity of phenotypes was observed and documented in Rice4619 (Figure S3, Supporting Information), encompassing a range of traits such as plant height, tiller angle and creeping growth, lesion mimics, dense panicle, fertility, grain size, flowering time, germination vigor, and tiller number. Among the mutant lines with visible phenotypes, we noticed a M2 line named A2038 that exhibited an early flowering phenotype under long‐day conditions. In a F2 segregating population (backcrossed to the wild‐type ZH11), we observed 109 normal plants and 32 early‐flowering plants, indicating a 3:1 ratio (goodness‐of‐fit test, *χ*2 < 0.05), which suggests a recessive mutation underlies the early‐flowering phenotype.

To identify the mutation responsible for this early flowering phenotype in A2038, we screened the Rice4619 re‐sequencing data and found a mutation at the 264th Glu in *Photoperiod Sensitivity 5* (*OsSE5*, *OsZH11G0613978000*) leading to premature translational termination (Figure S4, Supporting Information), which aligned well with the reported phenotype.^[^ ^15^ ^]^ We also identified *Wx* (*GBSSI*, *OsZH11G0611685100*) alleles in Rice4619, including an Gly‐to‐Asp substitutions at the 67th positions and an Arg‐to‐His substitution at the 483rd position. Interestingly, Arg483His mutations led to waxy endosperm phenotype (Figure S5, Supporting Information). Detailed descriptions of all such mutants are available for distribution via the CROPTILLING database (http://www.croptilling.com/ems/ZH11, Figure S6, Supporting Information). Simultaneously, we have also completed SNP calling analyses for the Rice 3K Genomes Project (named Rice3010 hereafter)^[^ ^16^ ^]^ using published next‐generation sequencing (NGS) data of 2800 varieties against the ZH11 reference genome^[^ ^10^ ^]^ and the same calling parameters as used for Rice4619. This analysis identified 11569585 SNPs, including 2355047 in genic regions, 1001562 within 1 Kb upstream, 691724 within 1 Kb downstream, 766503 within 1–2 Kb upstream, 540030 within 1–2 Kb downstream, and 6214719 intergenic SNPs located more than 2 Kb from gene bodies.

SNPs in Rice4619 were less enriched in pericentromeric regions compared with the SNP patterns in 2800 Asian rice varieties, with a higher frequency of mutations in gene‐dense regions and fewer in repetitive DNA regions (see Figure 1A for chromosomes 1, 2, and 3 and Figure S2, Supporting Information for all 12 chromosomes). However, analyses of SNPs and SNP^m^s in 100 Kb bins across the entire genome revealed that heterozygous SNP^m^s showed a better correlation than homozygous ones (Figure S7, Supporting Information). Among all identified SNP^m^s, 29.6% (EMS75) and 32.2% (EMS80) were located in genic regions, 22% (EMS75) and 22.3% (EMS80) were located in gene‐proximal regions (2 Kb upstream and downstream of genic regions), while the remaining 48.4% (EMS75) and 45.5% (EMS80) were found in intergenic regions (>2 Kb from genic regions, Figure 1C). No obvious differences were observed between homozygous and heterozygous SNP^m^s. Among 2.3 million SNP^m^s identified in genic regions, 30.5% were missense mutations, 0.54% affected splicing, 1% resulted in premature terminations in translation, 0.045% caused loss of stop codons, and the remaining 67.9% were synonymous (including 15.8% in coding regions and 52.1% in noncoding regions, namely, 5′UTRs, 3′UTRs, and introns; Figure 1D). We estimated that >97% of genes in the rice genome harbored one or more missense mutations (Figure 1E).

Compared with SNP distributions in Rice3010, the major difference was that many more SNP^m^s occurred in genic regions in Rice4619, for both EMS75 and EMS80 (Figure 1F). Additionally, the frequency of heterozygous SNP^m^s was higher than that of homozygous ones, further suggesting differential selection pressure. The proportion of missense mutations was also higher in Rice4619 (with an average of 2.6:1 (heterozygous) and 1.9:1 (homozygous) for missense: synonymous compared with 1.6:1 in Rice3010) (Figure 1G). We also analyzed SNP distribution patterns in other populations including 1001 *Arabidopsis* ecotypes (At1001; 1.7:1)^[^ ^13^ ^]^ and 2504 human individuals (Hs2504; 1.8:1),^[^ ^14^ ^]^ and observed similar missense mutation frequencies as in Rice3010 (Figure 1G). This suggests that compared with heterozygous SNP^m^s in Rice4619, which may have experienced relatively weaker selection pressures than those in natural populations, homozygous SNP^m^s may have undergone some degree of purifying selection due to lethal mutations.^[^ ^17^ ^]^

It has been proposed that EMS attacks the O‐6 group of guanine (*G*) on DNA to generate an alkylated guanine (*G*), and the biased alkylated G pairs with thymine (T) instead of cytosine (C), resulting in a G/C‐to‐A/T conversion during DNA replication.^[^ ^18^ ^]^ To characterize the mutational spectrum, we profiled all SNP^m^s in the Rice4619 mutant population. As expected, G/C‐to‐A/T conversions were the most prevalent mutation type in both Rice4619 and Rice3010. Notably, Rice4619 exhibited significantly higher G/C‐to‐A/T conversion rates among homozygous SNP^m^s, reaching 86.6% in EMS75 and 88.8% in EMS80, than among heterozygous SNP^m^s, which showed rates of 64.58% (EMS75) and 67.28% (EMS80).

To investigate the basis of this difference, we compared the functional consequences of G/C‐to‐A/T versus non‐G/C‐to‐A/T conversions. Non‐synonymous mutations resulting from non‐G/C‐to‐A/T conversions accounted for a higher proportion, ≈19.97% in EMS80 and 17.92% in EMS75, compared with 17.42% in EMS80 and 16.82% in EMS75 for G/C‐to‐A/T conversions (Table S6, Supporting Information). Similarly, non‐G/C‐to‐A/T conversions were associated with a greater increase in nonsense mutations (premature stop codons), showing a rise of 0.85% in EMS80 compared with an increase of 0.55% for G/C‐to‐A/T conversions.

Furthermore, BLOSUM62‐based scoring^[^ ^19^ ^]^ revealed that the average substitution score for amino acid changes induced by non‐G/C‐to‐A/T conversions was −3.07 in EMS80, which was markedly lower than the average score of −0.17 for G/C‐to‐A/T conversions (Table S7, Supporting Information). This indicates that non‐G/C‐to‐A/T conversions are more likely to generate deleterious protein‐altering mutations.

We therefore speculated that the elevated proportion of G/C‐to‐A/T conversions among homozygous SNP^m^s may result from purifying selection: deleterious mutations caused by non‐G/C‐to‐A/T conversions are more likely to be eliminated during the development of homozygous lines, leading to relative enrichment of G/C‐to‐A/T conversions in the surviving homozygous population.

**Figure 2:**
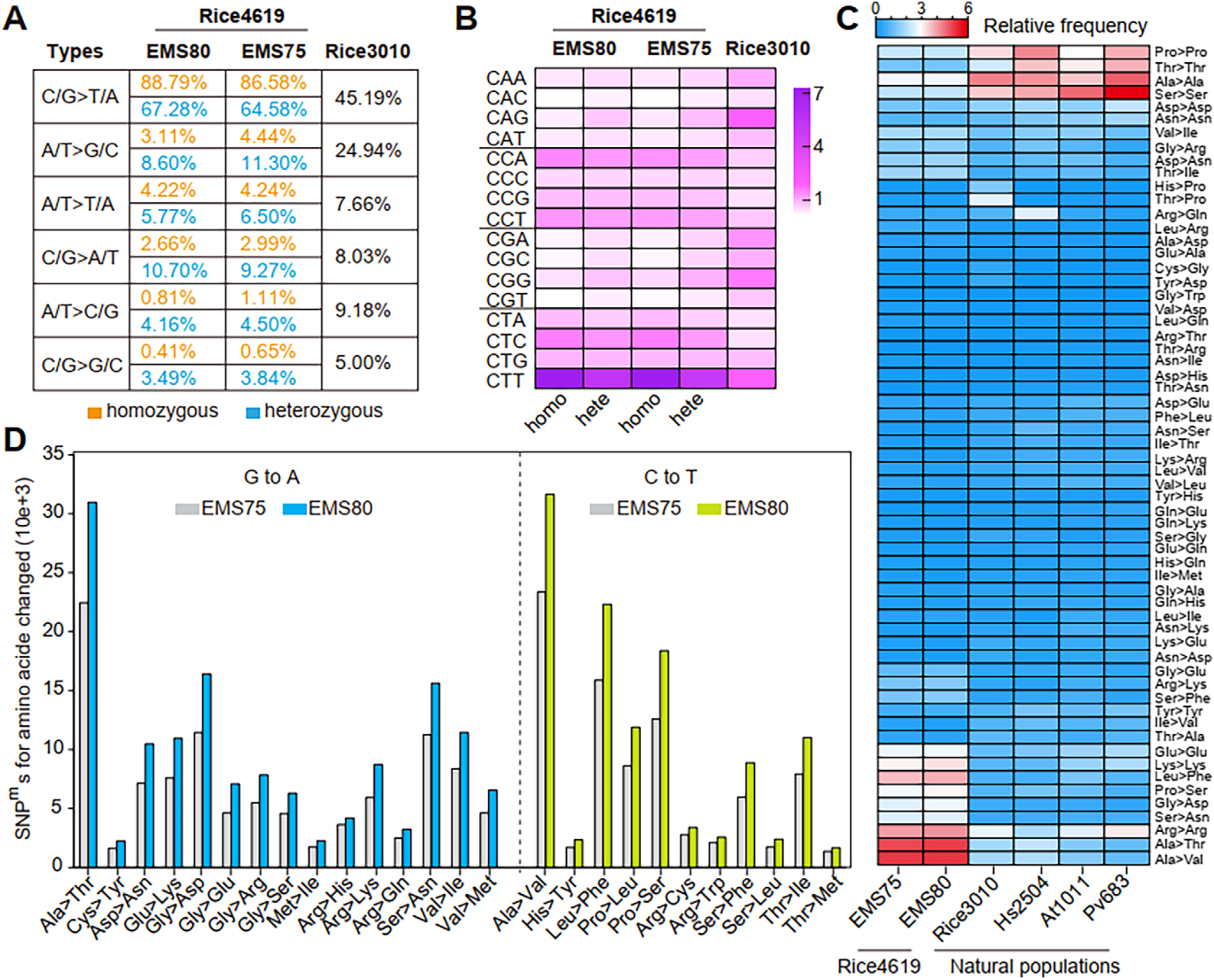
Nucleotide and amino acid substitutions in Rice4619. A) Comparison of nucleotide substitution types in Rice4619 and Rice3010. Notably, Rice4619 exhibits much higher frequencies of G/C‐to‐A/T substitutions and lower frequencies of T/A‐to‐G/C substitutions than Rice3010. B) Normalized SNP^m^ frequencies in different triplet contexts in Rice4619 and Rice3010. Mutations in CTN triplets (N = A, T, G, or C) are more frequent in Rice4619, whereas mutations in CGN triplets are enriched in Rice3010. C) Heatmap showing the percentages of various amino acid substitutions in Rice4619 and natural populations including Rice3010, Hs2504, At1001, and Pv683 (*Phaseolus vulgaris* L.,^[^ ^20^ ^]^). Note the elevated frequencies of Ala‐to‐Thr and Ala‐to‐Val substitutions in Rice4619, which are not observed in natural populations. D) Frequencies of amino acid substitutions specifically caused by G‐to‐A or C‐to‐T mutations. Ala‐to‐Thr substitutions are frequently caused by G‐to‐A changes, whereas Ala‐to‐Val substitutions predominantly arise from C‐to‐T mutations. Homo, homozygous SNP^m^s; Hete, heterozygous SNP^m^s.

Although G/C‐to‐A/T conversions were also the predominant mutation type in Rice3010 (**Figure** **2**A), this pattern may similarly reflect purifying selection. However, given that Rice3010 harbors spontaneous mutations derived from diverse endogenous processes, we cannot rule out the contribution of different mutational mechanisms to this spectrum. Thus, while a similar enrichment pattern is observed, its underlying causes may differ between EMS‐induced and spontaneous mutation populations.

We then examined SNP^m^s/SNPs in all triplet combinations. CTHs (where H represents A, T, or C) and CCNs (except CCC) were enriched in Rice4619 (for both EMS75 and EMS80, Figure 2B), while CGNs were enriched in Rice3010 (Figure 2B). Ala‐to‐Thr and Ala‐to‐Val conversions were most frequently observed in Rice4619, followed by Leu‐to‐Phe conversions. Such a pattern was not visible in Rice3010, Hs2504, At1001, and Pv683 (Figure 2C). We classified the frequencies of nucleotide substitutions with amino acid changes detected. As expected, frequent conversions of Ala‐to‐Thr and Ala‐to‐Val were caused primarily by G‐to‐A and C‐to‐T substitutions, respectively (Figure 2D).

### 2.2 SNP^m^s Occur Slightly More Often in Non‐Methylated Cs

**Figure 3:**
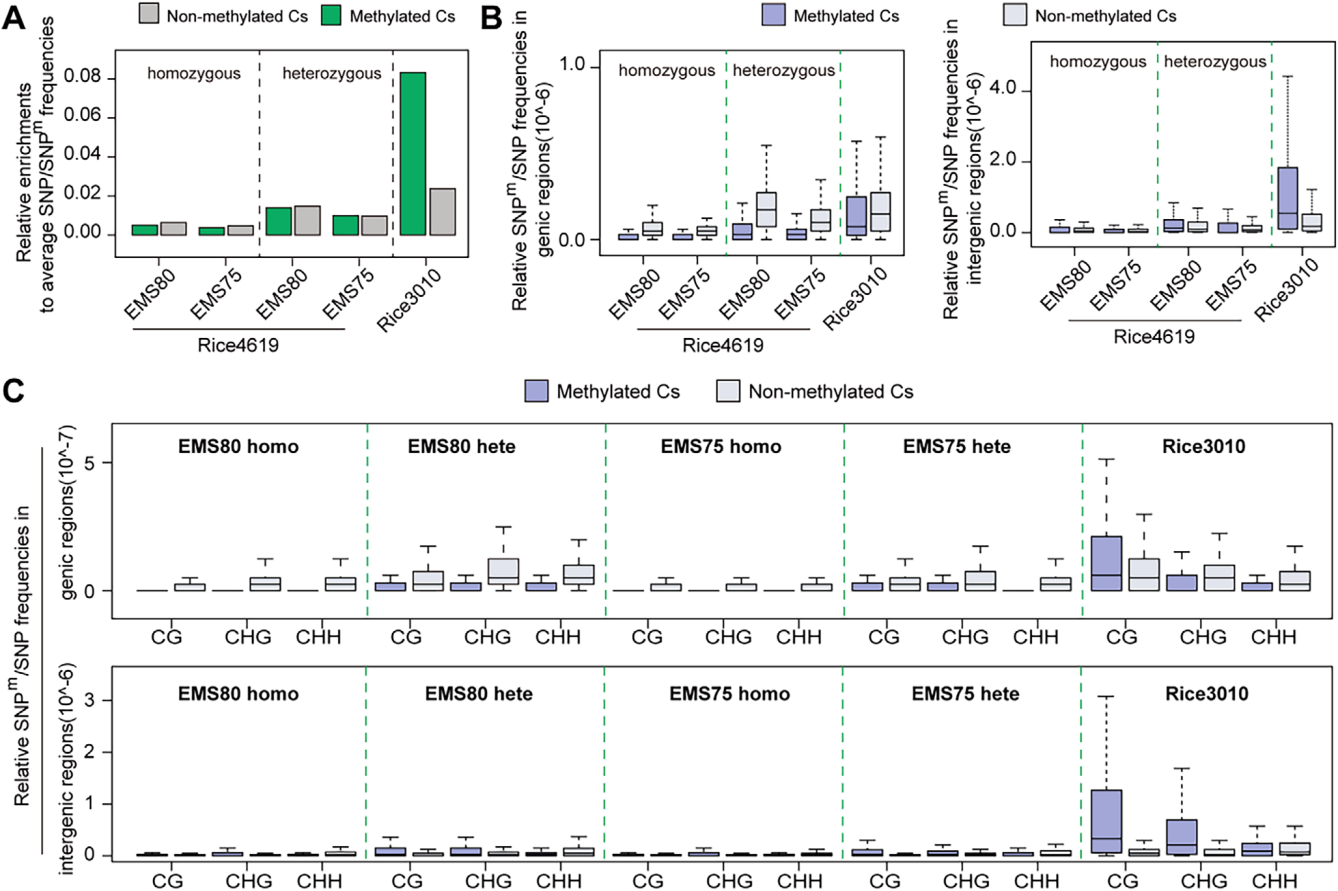
Impacts of C methylations on SNP^m^s and SNPs in Rice4619 and Rice3010. A) Relative SNP^m^/SNP frequencies at methylated and non‐methylated Cs. In Rice4619, SNP^m^s occur slightly more frequently at non‐methylated Cs than at methylated Cs, whereas in Rice3010, SNPs are markedly more enriched at methylated Cs. B) Relative SNP^m^/SNP frequencies at methylated and non‐methylated Cs in genic (left) and intergenic (right) regions. C) Relative SNP^m^/SNP frequencies in different methylation contexts, namely, CG, CHG, and CHH (where H represents A, T, or C), in genic (upper) and intergenic (lower) regions in Rice4619 and Rice3010. Note the consistently higher SNP frequencies at methylated CG in Rice3010. Homo, homozygous SNP^m^s; Hete, heterozygous SNP^m^s.

Methylated Cs in DNA, in particular 5mC, are broadly distributed in plant genomes and are reported to harbor more SNPs in natural populations.^[^ ^21^ ^]^ To investigate whether this is also the case in EMS‐induced mutagenesis, we compared SNP^m^s with the results of whole‐genome bisulfate sequencing.^[^ ^22^ ^]^ SNP^m^s in Rice4619 occurred more often in genic non‐methylated than methylated Cs, while in Rice3010, the number of SNPs in methylated Cs was 3.5‐fold higher than that in non‐methylated Cs (**Figure** **3**A). A detailed analysis of mutation patterns in genic and intergenic regions revealed that mutation rates were higher in non‐methylated Cs in genic regions for Rice4619. By contrast, for Rice3010, SNPs were particularly enriched in methylated Cs in intergenic regions (Figure 3A,B). The increased SNPs in methylated Cs of genic regions in Rice3010 were observed in the CG context, but not in the CHG and CHH contexts (CG, CHG, and CHH, where H represents A, T, or C; Figure 3C). However, genic and intergenic regions exhibited distinct patterns: in intergenic regions, SNP rates were higher in methylated Cs across CG and CHG (Figure 3C). In the Rice4619 population, higher SNP^m^ rates were observed in genic non‐methylated Cs (Figure 3C). Although the overall mutation pattern of heterozygous SNP^m^s resembles that of homozygous SNP^m^s, their genic distributions differ. The greater abundance of heterozygous SNP^m^s likely reflects the higher tolerance of heterozygosity for various mutation types. These results suggest that EMS‐induced mutations preferentially occur in genic non‐methylated Cs, with a bias toward CG, CHH and CHG sequence contexts, and the rates differ between genic and intergenic regions. The differences between Rice4619 and Rice3010 may be attributable to the weaker functional impact of mutations in methylated Cs, influenced by selective pressures during evolution and domestication. Taken together, these results suggest there is a fundamental difference between SNP^m^s in mutagenesis and SNPs in natural variations in relation to epigenomes.

### 2.3 Chemical‐Induced SNP^m^s are Preferentially Enriched in Accessible Chromatin Regions (ACRs)

**Figure 4:**
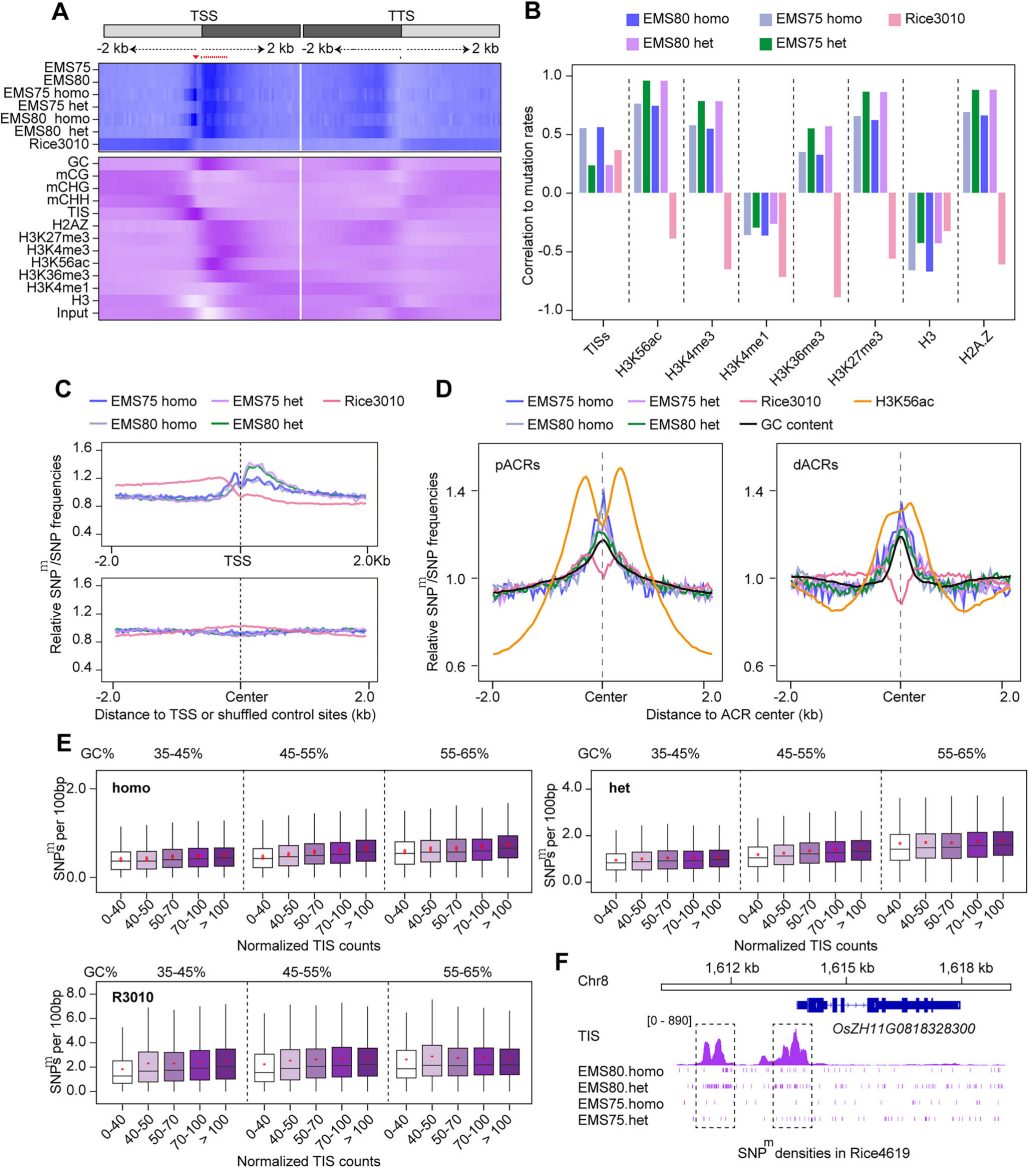
Associations between SNP^m^ frequencies and the chromatin status. A) Associations of SNP^m^/SNP frequencies with different genomic features. The average enrichment values were calculated in 8 Kb windows centered on genes, presented in 40 bp bins. TSS, transcription start site; TTS, transcription termination site; TIS, Tn5 transposome integration site. Darker shading represents higher frequencies. Note the elevated SNP^m^ frequencies (homozygous and heterozygous) and TIS counts in the 100 bp region upstream of TSSs (red arrowhead) and downstream of TSS regions (red dotted line) that also showed elevated GC contents, while in Rice3010, elevated SNP frequencies were only detected in 5′ upstream and 3′ downstream regions. B) Correlation analyses between SNP^m^/SNP frequencies and TISs, as well as various epigenetic modifications in Rice4619 (EMS75 and EMS80) and Rice3010. Positive correlations were detected between SNP^m^s and TISs (only in homozygous), H3K56ac, H3K4me3, H3K36me3, H3K27me3, and H2A.Z in Rice4619. C) Metaplot showing the SNP^m^/SNP distributions around TSSs (upper) and shuffled intergenic regions as a control (lower) in Rice4619 (EMS75 and EMS80) and Rice3010. SNP^m^ frequencies were elevated upstream (homozygous) and downstream (heterozygous) of TSSs in Rice4619, but not in Rice3010. D) SNP^m^/SNP frequencies in relation to GC contents and H3K56ac modifications in pACRs (left) and dACRs (right). Elevated SNP^m^ frequencies and GC contents were detected around centers of both pACRs and dACRs. E) Relative SNP^m^ frequencies in regions with different TIS counts and varying GC contents: 35–45% (left), 45–55% (middle), and 55–65% (right). Note that increased SNP^m^ frequencies were observed when TIS counts were increased. In all GC content groups, SNP^m^ frequencies increased with TIS counts, indicating that increased SNP^m^ levels were not merely due to higher GC contents. F) *OsGAPDH* (*OsZH11G0818328300*) as an example to show that SNP^m^s and TIS counts were higher in the region before the TSS (framed). Homo, homozygous SNP^m^s; Het, heterozygous SNP^m^s.

To determine whether EMS‐induced SNP^m^s are associated with specific genomic regions and chromatin states, we investigated chromatin accessibility using the Accessibility Assay using Transposase‐Generated Tags and Sequencing (ATAC‐seq) (Figure S8, Supporting Information) and mapped SNP^m^ frequencies in Rice4619 with various genomic and chromatin features. Homozygous SNP^m^s were most frequently enriched within 100 bp regions upstream of TSSs (**Figure** **4**A, red arrowhead), while heterozygous SNP^m^s showed increased frequencies both upstream and downstream of TSSs in both EMS75 and EMS80 lines (Figure 4A, red dotted line). The pattern of fewer homozygous SNP^m^s downstream of TSSs reflects the effects of purifying selection because mutations within gene body regions are more likely to be deleterious. By contrast, SNPs in the Rice3010 population were more enriched in upstream regions further than 100 bp from TSSs and in downstream regions of Transcription Termination Sites (TTSs) (Figure 4A).

The 100 bp regions upstream of TSSs are typically regarded as core promoters^[^ ^23^, ^24^ ^]^ and marked by enriched Tn5 transposome integration sites (TISs) in ATAC‐seq data, indicative of open chromatin.^[^ ^25^, ^26^ ^]^ Furthermore, the region downstream of a TSS is characterized by a higher GC content and is enriched with euchromatic histone marks, including H2A.Z, H3K27me3, H3K4me3, H3K36me3, and H3K56ac^[^ ^22^, ^25^, ^26^ ^]^ (Figure 4A). Association analyses using metaplots of average signal enrichment around genes revealed positive correlations between SNP^m^ densities in Rice4619 (both EMS75 and EMS80) and euchromatic histone modifications. Notably, only homozygous SNP^m^s (EMS75 and EMS80) were positively correlated with ATAC‐seq TIS signals (Figure 4B). By contrast, in Rice3010, the SNP frequency was negatively correlated with these epigenetic modification markers (Figure 4B).

The enrichment of SNP^m^s with TISs was only evident in genic regions, especially for homozygous SNP^m^s, and was not observed when shuffled (random) intergenic sequences were used (Figure 4C). We then plotted SNP^m^ frequencies with proximal ACRs (pACRs, located in genic and gene‐proximal regions) and distal ACRs (dACRs, located 2 Kb away from genic regions). SNP^m^ enrichment was observed in both cases for EMS75 and EMS80 in Rice4619 (Figure 4D), while no such enrichment was observed for SNPs in Rice3010 (Figure 4D, pink lines). Although H3K56ac is enriched around pACRs and dACRs, no distinct peak at the center was observed (Figure 4D, yellow lines).

GC contents are higher in ACRs (black line, Figure 4D)^[^ ^27^ ^]^ and SNP^m^s occur mostly at G/C sites (Figure 2A); therefore, we then investigated whether the elevated SNP^m^ frequencies are caused by higher GC contents. We divided ACRs into three groups based on GC contents, namely, 35–45%, 45–55%, and 55–65%, and further divided ACRs in each group into six subgroups according to their TIS counts: 0–10, 10–15, 15–20, 20–30, 30–60, and >60. For all three groups, the frequencies of both heterozygous and homozygous SNP^m^s were higher in regions with higher TIS counts (Figure 4E), suggesting that higher homozygous and heterozygous SNP^m^ frequencies in ACRs are not caused by higher GC contents. Taking the rice *glyceraldehyde‐3‐phosphate dehydrogenase* gene (*OsZH11G0818328300, OsGAPDH*) as an example, a large number of SNP^m^s was detected in the ACR (with higher TISs) located upstream of the TSS (Figure 4F, framed).

To further validate the observed bias in EMS‐induced mutations in rice, we extended our analysis to EMS/ENU‐induced mutations in *Caenorhabditis elegans* (The Million Mutation Project^[^ ^28^, ^29^ ^]^). Our findings were consistent with those in rice, showing that homozygous mutations are more frequently found in the 100 bp regions upstream of TSSs (Figure S9, Supporting Information).

In addition, we observed reduced enrichment of homozygous SNP^m^s downstream of TSSs compared with heterozygous SNP^m^s, suggesting the action of purifying selection. This implies that variations in *cis*‐regulatory elements (CREs) are more likely to be retained during mutagenesis, potentially contributing to the creation of new genetic materials that are often overlooked in EMS‐induced mutagenesis studies.

### 2.4 SNP^m^ Frequencies Positively Correlate with Gene Expression Levels

**Figure 5:**
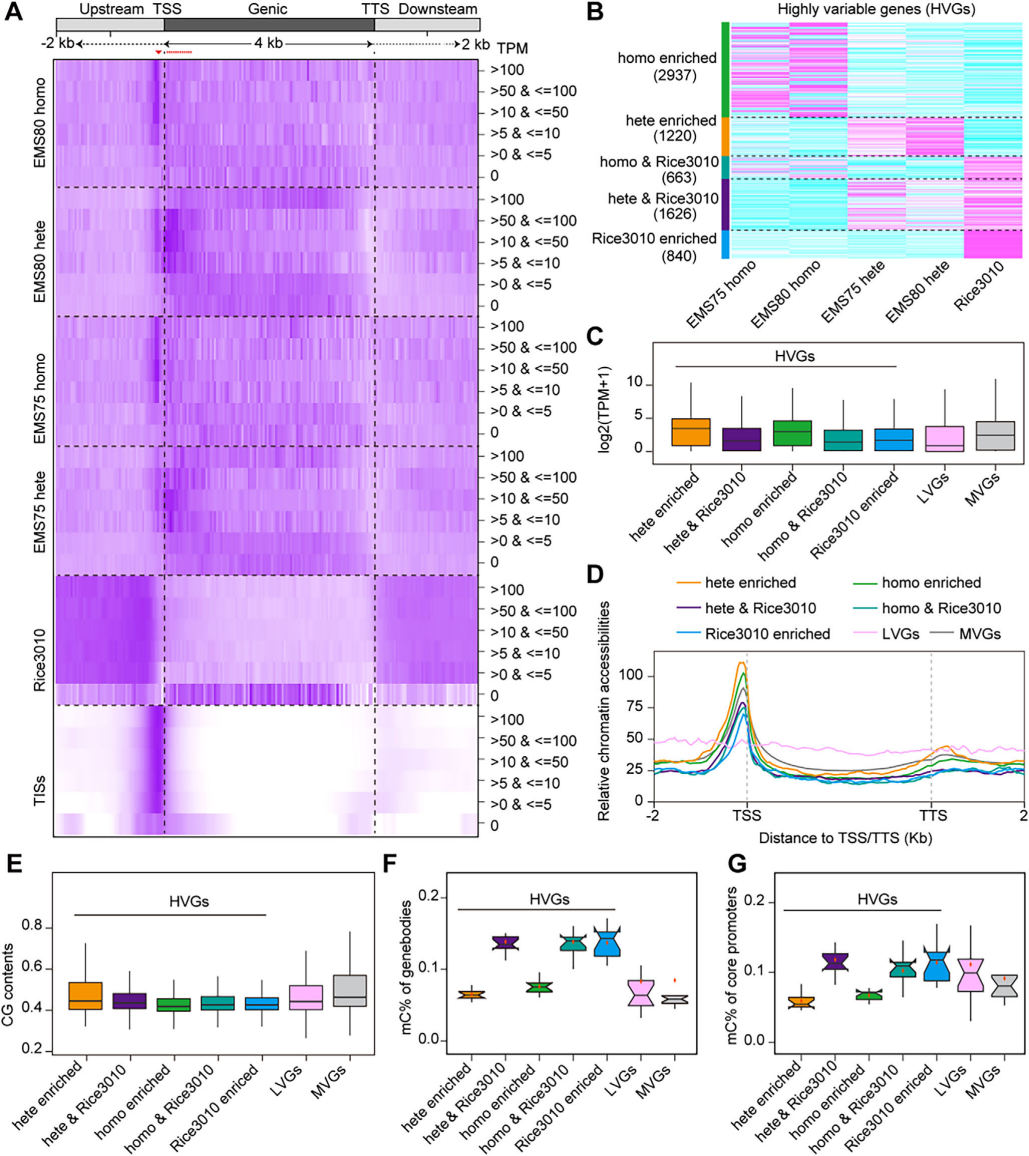
SNP^m^s are more prevalent in actively expressed genes. A) Distributions of SNP^m^s/SNPs in genomic regions with different levels of gene expression. All rice genes (excluding TEs) were divided into six groups according to their expression levels, and 40 bp bins were used and normalized to 8 Kb regions. Note that elevated homozygous SNP^m^ and TIS frequencies were observed in Rice4619 (EMS75 and EMS80) in a small region before TSSs (red arrowhead), while heterozygous SNP^m^s were enriched before and after TSSs (red dotted line) in genes with FPKM >5, both not in genes with FPKM ≤ 5 or silenced genes. Such a trend was not observed in Rice3010. B) Heatmap with hierarchical clustering showing the distribution of HVGs between Rice4619 and Rice3010. Note that Rice4619‐enriched HVGs in EMS75 and EMS80 were similar, but different from Rice3010‐enriched ones. C) Boxplots comparing the expression levels of genes in different variable groups. HVGs enriched (homozygous) in Rice4619 (both heterozygous and homozygous) displayed significantly higher expression than Rice3010‐enriched HVGs as well as LVGs and MVGs. D) Relative chromatin accessibilities (based on TIS counts) across gene groups. Rice4619‐enriched HVGs (including heterozygous enriched and homozygous enriched) showed substantially higher chromatin accessibilities than shared HVGs, MVGs, and Rice3010‐enriched HVGs. No obvious trend was observed for LVGs. E) GC content across gene variability groups. HVGs enriched in Rice4619 had significantly lower GC contents than those enriched in Rice3010 as well as LVGs and MVGs. F,G) Boxplots of C methylation (mC) percentages in genic (F) and core promoter (G) regions across different gene groups. Rice4619‐enriched HVGs (including heterozygous enriched and homozygous enriched) exhibited lower C methylation levels than Rice3010‐enriched ones for both genic and core promoter regions. Homo, homozygous SNP^m^s; Hete, heterozygous SNP^m^s.

Building on the observation that SNP^m^s are enriched in ACRs, we hypothesized that actively expressed genes may be enriched with SNP^m^s. We classified all genes expressed in leaves of 7‐day‐old rice seedlings^[^ ^22^ ^]^ into six groups using fragments per kilobase of transcripts per million fragments mapped (FPKM) to define their relative expression levels, and plotted homozygous and heterozygous SNP^m^s in genic and proximal regions. We then analyzed the distributions of homozygous and heterozygous SNP^m^s in genic and proximal regions. Heterozygous SNP^m^s were enriched in both upstream and downstream regions around TSSs, while homozygous SNP^m^s were predominantly enriched in upstream genic regions of genes with FPKM >5 for both EMS75 and EMS80 (**Figure** **5**A). Correspondingly, elevated TIS counts were also observed for genes with FPKM >5 (Figure 5A), but not for genes with FPKM ≤ 5 or silenced genes. By contrast, SNP enrichments for Rice3010 were observed in the 5′ upstream (before TSSs) and 3′ downstream regions of all expressed genes, regardless of the expression level (Figure 5A). Moreover, natural SNPs tend to be more frequently found in the 5′ upstream and 3′ downstream regions and at methylated Cs^[^ ^30^ ^]^ (Figure 3A and 4A), which may reflect the effects of long‐term natural selection that eliminates deleterious mutations in open chromatin and highly expressed genes, unlike EMS‐induced SNP^m^s (Figure 5A), which have not undergone such evolutionary filtering.

We then grouped all genes in the rice genome based on their SNP^m^s/SNPs per gene per million mutations (SPGM), which allowed us to identify 7286 highly variable genes (HVGs, with a cutoff of SPGM >50 in at least one population of EMS75, EMS80, and Rice3010), 1057 lowly variable genes (LVGs, with a cutoff of SPGM < 5), and the remaining 28995 as moderately variable genes (MVGs). Among the HVGs, 1220 were enriched in heterozygous SNP^m^s, indicating a high mutation frequency but stronger purging effects, and 2937 were enriched in homozygous SNP^m^s, suggesting a high mutation frequency with weaker purging effects (Figure 5B). Additionally, 663 HVGs were enriched in Rice3010 with homozygous SNP^m^s, while 1626 were enriched in Rice3010 with heterozygous SNP^m^s (Figure 5B). Finally, 840 HVGs were specifically enriched in Rice3010 (Figure 5B). By plotting their expression levels, we observed that homozygous and heterozygous enriched HVGs were more likely to be actively expressed genes (Wilcoxon rank test, *p* < 2.1e^−16^), with average expression levels much higher than Rice3010‐related HVGs, LVGs, and MVGs (Figure 5C). Heterozygous enriched HVGs had higher chromatin accessibilities than Rice3010‐related HVGs, LVGs, and MVGs (Figure 5D). The average GC content in HVGs was lower than those in LVGs and MVGs (Figure 5E), suggesting further that the higher frequency of SNP^m^s in HVGs was not caused by higher GC contents. Furthermore, it is noteworthy that Rice3010‐enriched HVGs had higher DNA methylation levels than Rice4619‐enriched ones, for both genic (Figure 5F) and core promoter (−200 to + 50 bp of TSSs, Figure 5G) regions, which is in agreement with the observed higher level of SNPs in methylated Cs in Rice3010 (Figure 3A). Gene ontology (GO) analysis revealed that HVGs from both Rice3010 and Rice4619 were enriched in pathways related to signal transduction, ADP binding, chromosome organization, and oxidoreductase activity (Tables S8 and S9, Supporting Information), implying a potential adaptive strategy in plants.

In summary, our study demonstrates that EMS‐induced mutations preferentially occur in the CTH and CCN (except CCC) context, ACRs, and actively expressed genes, while showing relative insensitivity to DNA methylation. Moreover, the significant overlap observed between EMS‐induced SNP^m^s and natural SNPs suggests a potential adaptive mechanism is employed by plants.

## 3 Discussion

Although EMS‐induced mutagenesis is often assumed to introduce random mutations, our genome‐wide profiling of over 7 million SNP^m^s in 4619 rice mutants revealed clear biases toward ACRs and actively transcribed genes. SNP^m^s were highly enriched near TSSs, particularly within ≈100 bp upstream and downstream. These regions contain many TISs and DNase I‐hypersensitive sites, suggesting that open chromatin is more susceptible to chemical mutagens.^[^ ^25^, ^31^, ^32^ ^]^

Homozygous SNP^m^s were less frequent in gene body regions downstream of TSSs, especially in highly expressed genes (FPKM >5), indicative of purifying selection against deleterious coding mutations during plant development. By contrast, heterozygous SNP^m^s were more broadly distributed, likely due to partial masking of detrimental effects when one functional allele remains. This difference reflects early‐stage selection acting on mutation fate, even within a few generations of self‐pollination.

It is plausible that actively expressed genes are more likely located in ACRs, which offer greater accessibility to mutagens, resulting in more mutations compared to tightly packed chromatin regions where genes are less actively expressed.^[^ ^32^, ^33^ ^]^ Previous studies have shown that the small region upstream of TSSs (5′ upstream) contains abundant TISs and DNaseI hypersensitive sites.^[^ ^22^, ^32^ ^]^ In humans, pluripotent and immortalized cells exhibit higher mutation rates than differentiated cells.^[^ ^32^ ^]^ Furthermore, different chromatin states exhibit variable efficiencies in mutagenesis during genome editing and DNA double‐strand break repair.^[^ ^34^ ^]^

*Cis*‐regulatory variants, which are often located upstream of TSSs,^[^ ^24^ ^]^ appear to be less deleterious and thus more likely to persist.^[^ ^23^, ^31^, ^35^ ^]^ This is exemplified by an EMS‐induced SNP^m^ near the *OsNRT2.3* TSS that enhances grain yield.^[^ ^36^ ^]^ Such regulatory mutations are frequently overlooked but represent a valuable source of phenotypic variation.

Although the distribution of EMS‐induced mutations is strongly influenced by chromatin structure, natural mutation patterns are modulated by chromatin features such as DNA methylation, histone modifications, and accessibility.^[^ ^30^ ^]^ However, the difference in observed patterns between natural and EMS‐induced SNPs is likely shaped by long‐term purifying selection acting on natural mutations. Deleterious mutations in expressed genes and open chromatin regions are selectively removed over time, resulting in the apparent depletion of natural SNPs in those regions. By contrast, EMS‐induced mutations are recent and reflect the primary bias of mutation occurrence before selection has acted, revealing a direct association with open chromatin and active transcription.

Together, these findings demonstrate that EMS‐induced mutations are not randomly distributed but are influenced by chromatin structure and gene expression. This chromatin‐driven mutational bias, coupled with early purifying selection, shapes the spectrum of observable SNP^m^s. Our work highlights the importance of noncoding regulatory regions in functional genomics and breeding, and provides a high‐resolution mutant resource (www.croptilling.com/ems/ZH11) for dissecting both gene and CRE function in crops.

## 4 Experimental Section

### Mutagenesis and Generation of Rice4619

Seeds of the *japonica* rice variety ZH11 were mutagenized with 70, 75, and 80 mm EMS as described previously.^[^ ^33^ ^]^ Briefly, ≈20000 seeds per treatment were pre‐soaked in water at 25 °C for 16 h, transferred to different concentrations of EMS in 0.1 m phosphate‐buffered saline (pH 7.1) in 2‐L flasks, sealed with cling film, shaken every 30 min for 24 h, washed ten times with distilled water, and then incubated in distilled water for another 24 h. These mutagenized M1 seeds were used for germination tests (Table S1, Supporting Information) and then sowed in the field. M2 seeds were harvested from fertile M1 plants following 75 and 80 mm EMS treatments after self‐fertilization, and eight M2 seeds were sowed from lines with sufficient seeds to generate M2 populations. M3 seeds were harvested from M2 lines that produced sufficient seeds and given a unique serial number to generate the Rice4619 population. The number of M1 seeds germinated, fertile M1 plants produced, and M2 lines with an albino phenotype were used to estimate mutation frequencies (Table S1, Supporting Information). The subpopulation of EMS70 was discarded due to its low mutation frequency (Table S1, Supporting Information).

### DNA Extraction and Genomic Sequencing

Genomic DNAs were extracted from four seeds of ZH11 and M3 plants using the cetyl trimethyl ammonium bromide method and quantified using a PicoGreen dsDNA Assay Kit (P7581, Life Technologies, USA) with a microplate reader (Tecan Infinite M1000 PRO, Thermo Fisher Scientific, USA) to obtain at least 3 µg of genomic DNA from each line. The integrity of DNAs was assayed by electrophoresis using a 1% agarose gel, and low‐quality DNA samples were removed. Qualified samples were purified further using DNA magnetic beads (GO‐PCRC, GeneOn BioTech, China) and quantified using the Qubit system (Thermo Fisher Scientific). Libraries were constructed for each sample following the Illumina manual, and paired‐end 150‐bp sequencing was performed on a HiSeq X Ten sequencing system (Illumina, USA) in the Joint Genome Institute following the manufacturer's instructions. Raw sequences were processed to remove adaptors and low‐quality reads.

Examination of the effects of SNPs and SNP^m^s. Clean paired‐end 150‐bp reads of Rice3010 (2800 samples) and Rice4619 (4619 samples) were mapped to the ZH11 reference genome.^[^ ^10^ ^]^ and Nipponbare genome version 7^[^ ^37^ ^]^ using Burrows–Wheeler Aligner‐MEM (BWA version 0.7.15) with default parameters.^[^ ^38^ ^]^ The aligned reads were sorted, and duplicates were removed using Picard tools (release 2.9.0) (http://broadinstitute.github.io/picard). SNPs/SNP^m^s were called using the Genome Analysis Toolkit (GATK 3.7) and Sentieon tools^[^ ^39^, ^40^ ^]^ with minor modifications. Generally, GATK HaplotypeCaller was used to call SNP^m^s/SNPs with the parameter “‐stand_call_conf 10.” A joint genotyping step for comprehensive SNP/SNP^m^ union and filtering was performed in Rice4619 to generate the emit‐all‐sites variant call format (VCF) files. Common mutations detected in multiple samples were removed as SNPs and modified as described previously.^[^ ^41^ ^]^ Mutations with a read depth ≥ 3 were defined as valid. If a mutation occurred in more than ten individual accessions, it was removed. InDels within 10 bp in Rice4619 were also identified but not included in the following analysis. To define the distributions of SNP^m^s and SNPs in functional genes, 37338 (ZH11) genes (without TEs) were used for mutation bias analyses because TEs may have different epigenetic modifications and chromatin accessibilities.

SNP^m^s and SNPs in the genome were determined based on ZH11 reference annotation^[^ ^10^ ^]^ using Annovar^[^ ^42^ ^]^ and SnpEff.^[^ ^43^ ^]^ SNP^m^s distributions in line plots were generated using TBtools software.^[^ ^44^ ^]^

### ATAC‐seq

ATAC‐seq was performed following our previously established protocol.^[^ ^45^ ^]^ Fresh leaf tissue (1 g) of ZH11 was chopped in 1 mL of ice‐cold lysis buffer (15 mm Tris‐HCl pH 7.5, 20 mm NaCl, 80 mm KCl, 0.5 mm spermine, 5 mm 2‐mercaptoethanol, and 0.2% Triton X‐100) and filtered twice through a 40 µm filter. The DAPI‐stained nuclei were sorted on a BD FACSCanto flow cytometer and then collected by centrifugation and washed with Tris‐Mg buffer (10 mm Tris‐HCl pH 8.0 and 5 mm MgCl_2_). Tn5 transposomes in 40 µL of TTBL buffer (Vazyme, TD501) were added, followed by incubation at 37 °C for 30 min. After purification with a NEB Monarch DNA Cleanup Kit (T1030S), the products were amplified using NEB Next Ultra II Q5 Master Mix (M0544L) and purified with Hieff NGS DNA Selection Beads (Yeasen, 12601ES03).

### Identifications of ACRs

Histone ChIP‐seq data for rice leaves were downloaded from NCBI and processed as described previously.^[^ ^22^ ^]^ Raw reads were trimmed using Trimmomatic v.0.36^[^ ^46^ ^]^ and aligned to the ZH11 reference genome^[^ ^10^ ^]^ using Bowtie v.2.3.5^[^ ^47^ ^]^ with the following parameter: “‐X 1000 –vry‐sensitive”. Aligned reads with MAPQ < 10 were filtered out, and PCR duplicates were removed with Picard v.2.16.0 (http://broadinstitute.github.io/picard). ACRs were identified by MACS2^[^ ^48^ ^]^ using the parameter “–keep‐dup all –shift ‐100 –extsizes 200 –nomodel ‐g 3.8e8,” and histone modifications were identified using the parameter “–keep‐dup all –extsizes 200 –nomodel ‐g 3.8e8.”

### Metaplot Analysis

Frequencies of SNP^m^s, SNPs, and ATAC‐seq and histone ChIP‐seq data in 2 Kb upstream to 2 Kb downstream regions around ACR centers and annotated TTSs or genic regions were calculated using deepTools v.3.0.2^[^ ^49^ ^]^ with the parameter “‐a 2000 ‐b 2000 ‐bs 40.” The average value of each column of the output matrix was normalized to the average value of each bin, and normalized values were used to create metaplots.

### Identifications of Methylated and Non‐Methylated Cs

DNA methylation data for rice leaves were downloaded from NCBI^[^ ^22^ ^]^ and aligned to the ZH11 reference genome, and methylated sites were called using the methylpy pipeline.^[^ ^50^ ^]^ Reads that mapped to multiple locations and clonal reads were removed with SAMtools.^[^ ^51^ ^]^ The non‐conversion rate was calculated via reads mapped to the chloroplast genome. Cs were called as methylated using a binomial test with the non‐conversion rate as the expected probability, followed by multiple testing correction using the Benjamini–Hochberg false discovery rate. Other Cs covered by aligned reads were considered to be non‐methylated.

## Conflict Of Interest

The authors declare no conflict of interest.

## Author Contributions

X.F.Y., Y.L. contributed equally to this work. C.M.L. is solely responsible for distributions of materials described in this manuscript. C.M.L., Z.L., H.L., and X.F.Y. conceived the project. X.F.Y., Z.L., and Y.L. performed the majority of the experiments. G.Q.J. assisted in generating the EMS populations. X.F.Y., Z.Y.L., W.S.W., and H.L. collected the sequencing data. X.F.Y., Z.L., H.L., and Y.L. conducted data analysis. X.F.Y. and Z.L. drafted the manuscript, and C.M.L. finalized it. All authors contributed to the discussion of the results and reviewed and commented on the manuscript.

## Supporting information



Supporting Information

Supplementary Table S1–S6

Supplementary Table S7

Supplementary Table S8

Supplementary Table S9

## Data Availability

All sequenced data in this study are available through the China National Center for Bioinformation/Beijing Institute of Genomics (https://ngdc.cncb.ac.cn/gsa) and the Chinese Academy of Sciences under the BioProject numbers PRJCA020505 and PRJCA034433. Genotypic data against ZH11 for searching and browsing (JBrowse)^[^ ^52^ ^]^ are available from the CROPTILLING database at http://www.croptilling.com/ems/ZH11.

## References

[1] R. D. H. Barrett , D. Schluter , Trends Ecol. Evol. 2008, 23, 38. 18006185 10.1016/j.tree.2007.09.008

[2] K. Bomblies , C. L. Peichel , Proc. Natl. Acad. Sci. 2022, 119, 2122152119. 10.1073/pnas.2122152119 PMC9335183 35858399

[3] E. A. Greene , C. A. Codomo , N. E. Taylor , J. G. Henikoff , B. J. Till , S. H. Reynolds , L. C. Enns , C. Burtner , J. E. Johnson , A. R. Odden , L. Comai , S. Henikoff , Genetics 2003, 164, 731. 12807792 10.1093/genetics/164.2.731 PMC1462604

[4] D. J. Futuyma , Evolutionary Biology, 2nd ed. Sinauer Associates, Sunderland 1986.

[5] A. Farrell , B. I. Coleman , B. Benenati , K. M. Brown , I. J. Blader , G. T. Marth , M. J. Gubbels , BMC Genom. 2014, 15, 354. 10.1186/1471-2164-15-354 PMC4035079 24885922

[6] S. Gottwald , P. Bauer , T. Komatsuda , U. Lundqvist , N. Stein , BMC Res. Notes 2009, 2, 258. 20017921 10.1186/1756-0500-2-258 PMC2803498

[7] W. Yan , X. W. Deng , C. Yang , X. Tang , Front Plant Sci. 2021, 12, 579675. 33841451 10.3389/fpls.2021.579675 PMC8025102

[8] J. G. Monroe , T. Srikant , P. Carbonell‐Bejerano , C. Becker , M. Lensink , M. Exposito‐Alonso , M. Klein , J. Hildebrandt , M. Neumann , D. Kliebenstein , M. L. Weng , E. Imbert , J. Ågren , M. T. Rutter , C. B. Fenster , D. Weigel , Nature 2022, 602, 101. 35022609 10.1038/s41586-021-04269-6 PMC8810380

[9] L. Wang , A. T. Ho , L. D. Hurst , S. Yang , Nature 2023, 619, E52. 37495884 10.1038/s41586-023-06314-y PMC10371861

[10] P. Qin , H. Lu , H. Du , H. Wang , W. Chen , Z. Chen , Q. He , S. Ou , H. Zhang , X. Li , X. Li , Y. Li , Y. Liao , Q. Gao , B. Tu , H. Yuan , B. Ma , Y. Wang , Y. Qian , S. Fan , W. Li , J. Wang , M. He , J. Yin , T. Li , N. Jiang , X. Chen , C. Liang , S. Li , Cell 2021, 184, 3542. 34051138 10.1016/j.cell.2021.04.046

[11] J. C. Roach , G. Glusman , A. F. A. Smit , C. D. Huff , R. Hubley , P. T. Shannon , L. Rowen , K. P. Pant , N. Goodman , M. Bamshad , J. Shendure , R. Drmanac , L. B. Jorde , L. Hood , D. J. Galas , Science 2010, 328, 636. 20220176 10.1126/science.1186802 PMC3037280

[12] B. J. Till , J. Cooper , T. H. Tai , P. Colowit , E. A. Greene , S. Henikoff , L. Comai , BMC Plant Biol. 2007, 7, 19. 17428339 10.1186/1471-2229-7-19 PMC1858691

[13] D. Weigel , R. Mott , Genome Biol. 2009, 10, 107. 19519932 10.1186/gb-2009-10-5-107 PMC2718507

[14] P. H. Sudmant , T. Rausch , E. J. Gardner , R. E. Handsaker , A. Abyzov , J. Huddleston , Y. Zhang , K. Ye , G. Jun , M. H. Fritz , M. K. Konkel , A. Malhotra , A. M. Stütz , X. Shi , F. P. Casale , J. Chen , F. Hormozdiari , G. Dayama , K. Chen , M. Malig , M. J. P. Chaisson , K. Walter , S. Meiers , S. Kashin , E. Garrison , A. Auton , H. Y. K. Lam , X. J. Mu , C. Alkan , D. Antaki , et al., Science 2015, 526, 75.

[15] F. Andrés , D. W. Galbraith , M. Talón , C. Domingo , Plant Physiol. 2009, 151, 681. 19675157 10.1104/pp.109.139097 PMC2754645

[16] W. Wang , R. Mauleon , Z. Hu , D. Chebotarov , S. Tai , Z. Wu , M. Li , T. Zheng , R. R. Fuentes , F. Zhang , L. Mansueto , D. Copetti , M. Sanciangco , K. C. Palis , J. Xu , C. Sun , B. Fu , H. Zhang , Y. Gao , X. Zhao , F. Shen , X. Cui , H. Yu , Z. Li , M. Chen , J. Detras , Y. Zhou , X. Zhang , Y. Zhao , D. Kudrna , et al., Nature 2018, 557, 43. 29695866 10.1038/s41586-018-0063-9 PMC6784863

[17] I. P. Gorlov , M. Kimmel , C. I. Amos , Hum. Mol. Genet. 2006, 15, 1143. 16500998 10.1093/hmg/ddl029

[18] S. Ossowski , K. Schneeberger , J. I. Lucas‐Lledó , N. Warthmann , R. M. Clark , R. G. Shaw , D. Weigel , M. Lynch , Science 2010, 327, 92. 20044577 10.1126/science.1180677 PMC3878865

[19] S. Henikoff , J. G. Henikoff , Proc. Natl. Acad. Sci. 1992, 89, 10915. 1438297 10.1073/pnas.89.22.10915 PMC50453

[20] J. Wu , L. Wang , J. Fu , J. Chen , S. Wei , S. Zhang , J. Zhang , Y. Tang , M. Chen , J. Zhu , L. Lei , Q. Geng , C. Liu , L. Wu , X. Li , X. Wang , Q. Wang , Z. Wang , S. Xing , H. Zhang , M. W. Blair , S. Wang , Nat. Genet. 2020, 52, 118. 31873299 10.1038/s41588-019-0546-0

[21] D. Q. Shi , I. Ali , J. Tang , W. C. Yang , Front. Genet. 2017, 8, 100. 28769976 10.3389/fgene.2017.00100 PMC5515870

[22] Z. Lu , A. P. Marand , W. A. Ricci , C. L. Ethridge , X. Zhang , R. J. Schmitz , Nat. Plants 2019, 5, 1250. 31740772 10.1038/s41477-019-0548-z

[23] J. E. F. Butler , J. T. Kadonaga , Gene Dev. 2002, 16, 2583. 12381658 10.1101/gad.1026202

[24] U. Ohler , Nucleic Acids Res. 2006, 34, 5943. 17068082 10.1093/nar/gkl608 PMC1635271

[25] S. L. Klemm , Z. Shipony , W. J. Greenleaf , Nat. Rev. Genet. 2019, 20, 207. 30675018 10.1038/s41576-018-0089-8

[26] Q. Li , H. Zhou , H. Wurtele , B. Davies , B. Horazdovsky , A. Verreault , Z. Zhang , Cell 2008, 134, 244. 18662540 10.1016/j.cell.2008.06.018 PMC2597342

[27] J. Wang , J. Zhuang , S. Iyer , X. Lin , T. W. Whitfield , M. C. Greven , B. G. Pierce , X. Dong , A. Kundaje , Y. Cheng , O. J. Rando , E. Birney , R. M. Myers , W. S. Noble , M. Snyder , Z. Weng , J. Biomol. Struct. Dyn. 2013, 31, 49.

[28] S. Flibotte , M. L. Edgley , I. Chaudhry , J. Taylor , S. E. Neil , A. Rogula , R. Zapf , M. Hirst , Y. Butterfield , S. J. Jones , M. A. Marra , R. J. Barstead , D. G. Moerman , Genetics 2010, 185, 431. 20439774 10.1534/genetics.110.116616 PMC2881127

[29] O. Thompson , M. Edgley , P. Strasbourger , S. Flibotte , B. Ewing , R. Adair , V. Au , I. Chaudhry , L. Fernando , H. Hutter , A. Kieffer , J. Lau , N. Lee , A. Miller , G. Raymant , B. Shen , J. Shendure , J. Taylor , E. H. Turner , L. W. Hillier , D. G. Moerman , R. H. Waterston , Genome Res. 2013, 23, 1749. 23800452 10.1101/gr.157651.113 PMC3787271

[30] M. Tomkova , B. Schuster‐Bockler , Trends Genet. 2018, 34, 627. 29853204 10.1016/j.tig.2018.04.005

[31] H. Pei , Y. Li , Y. Liu , P. Liu , J. Zhang , X. Ren , Z. Lu , Abiotech 2023, 4, 8. 37220536 10.1007/s42994-023-00095-8 PMC10199822

[32] R. E. Thurman , E. Rynes , R. Humbert , J. Vierstra , M. T. Maurano , E. Haugen , N. C. Sheffield , A. B. Stergachis , H. Wang , B. Vernot , K. Garg , S. John , R. Sandstrom , D. Bates , L. Boatman , T. K. Canfield , M. Diegel , D. Dunn , A. K. Ebersol , T. Frum , E. Giste , A. K. Johnson , E. M. Johnson , T. Kutyavin , B. Lajoie , B. K. Lee , K. Lee , D. London , D. Lotakis , S. Neph , et al., Nature 2012, 489, 75. 22955617 10.1038/nature11232 PMC3721348

[33] C. R. Epigenomics , A. Kundaje , W. Meuleman , J. Ernst , M. Bilenky , A. Yen , A. Heravi‐Moussavi , P. Kheradpour , Z. Zhang , J. Wang , M. J. Ziller , V. Amin , J. W. Whitaker , M. D. Schultz , L. D. Ward , A. Sarkar , G. Quon , R. S. Sandstrom , M. L. Eaton , Y. C. Wu , A. R. Pfenning , X. Wang , M. Claussnitzer , Y. Liu , C. Coarfa , R. A. Harris , N. Shoresh , C. B. Epstein , E. Gjoneska , D. Leung , et al., Nature 2015, 518, 317.25693563

[34] T. Weiss , P. A. Crisp , K. M. Rai , M. Song , N. M. Springer , F. Zhang , Plant Physiol. 2022, 190, 1153. 35689624 10.1093/plphys/kiac285 PMC9516779

[35] H. Pei , W. Teng , L. Gao , H. Gao , X. Ren , Y. Liu , J. Jia , Y. Tong , Y. Wang , Z. Lu , Sci. China Life Sci. 2023, 66, 819. 36417050 10.1007/s11427-022-2202-3

[36] Y. Zhang , H. Tateishi‐Karimata , T. Endoh , Q. Jin , K. Li , X. Fan , Y. Ma , L. Gao , H. Lu , Z. Wang , A. E. Cho , X. Yao , C. Liu , N. Sugimoto , S. Guo , X. Fu , Q. Shen , G. Xu , L. R. Herrera‐Estrella , X. Fan , Sci. Adv. 2022, 8, adc9785. 10.1126/sciadv.adc9785 PMC9683703 36417515

[37] Y. Kawahara , M. de la Bastide , J. P. Hamilton , H. Kanamori , W. R. McCombie , S. Ouyang , D. C. Schwartz , T. Tanaka , J. Wu , S. Zhou , K. L. Childs , R. M. Davidson , H. Lin , L. Quesada‐Ocampo , B. Vaillancourt , H. Sakai , S. S. Lee , J. Kim , H. Numa , T. Itoh , C. R. Buell , T. Matsumoto , Rice 2013, 6, 4. 24280374 10.1186/1939-8433-6-4 PMC5395016

[38] H. Li , R. Durbin , Bioinformatics 2009, 25, 1754. 19451168 10.1093/bioinformatics/btp324 PMC2705234

[39] D. Freed , R. Aldana , J. A. Weber , J. S. Edwards , BioRxiv 2017, 10.1101/115717.

[40] K. I. Kendig , S. Baheti , M. A. Bockol , et al., Front. Genet. 2019, 10, 471268. 10.3389/fgene.2019.00736 PMC6710408 31481971

[41] G. Li , R. Jain , M. Chern , N. T. Pham , J. A. Martin , T. Wei , W. S. Schackwitz , A. M. Lipzen , P. Q. Duong , K. C. Jones , L. Jiang , D. Ruan , D. Bauer , Y. Peng , K. W. Barry , J. Schmutz , P. C. Ronald , Plant Cell 2017, 29, 1218. 28576844 10.1105/tpc.17.00154 PMC5502455

[42] K. Wang , M. Li , H. Hakonarson , Nucleic Acids Res. 2010, 38, e164. 20601685 10.1093/nar/gkq603 PMC2938201

[43] P. Cingolani , A. Platts , L. L. Wang , M. Coon , T. Nguyen , L. Wang , S. J. Land , X. Lu , D. M. Ruden , Fly 2012, 6, 80. 22728672 10.4161/fly.19695 PMC3679285

[44] C. Chen , H. Chen , Y. Zhang , H. R. Thomas , M. H. Frank , Y. He , R. Xia , Mol. Plant 2020, 13, 1194. 32585190 10.1016/j.molp.2020.06.009

[45] Z. Lu , B. T. Hofmeister , C. Vollmers , R. M. DuBois , R. J. Schmitz , Nucleic Acids Res. 2017, 45, 41. 10.1093/nar/gkw1179 PMC5389718 27903897

[46] A. M. Bolger , M. Lohse , B. Usadel , Bioinformatics 2014, 30, 2114. 24695404 10.1093/bioinformatics/btu170 PMC4103590

[47] B. Langmead , S. L. Salzberg , Nat. Methods 2012, 9, 357. 22388286 10.1038/nmeth.1923 PMC3322381

[48] Y. Zhang , T. Liu , C. A. Meyer , J. Eeckhoute , D. S. Johnson , B. E. Bernstein , C. Nusbaum , R. M. Myers , M. Brown , W. Li , X. S. Liu , Genome Biol. 2008, 9, R137. 18798982 10.1186/gb-2008-9-9-r137 PMC2592715

[49] F. Ramírez , F. Dündar , S. Diehl , B. A. Grüning , T. Manke , Nucleic Acids Res. 2014, 42, W187. 24799436 10.1093/nar/gku365 PMC4086134

[50] M. D. Schultz , Y. He , J. W. Whitaker , M. Hariharan , E. A. Mukamel , D. Leung , N. Rajagopal , J. R. Nery , M. A. Urich , H. Chen , S. Lin , Y. Lin , I. Jung , A. D. Schmitt , S. Selvaraj , B. Ren , T. J. Sejnowski , W. Wang , J. R. Ecker , Nature 2015, 523, 212. 26030523 10.1038/nature14465 PMC4499021

[51] H. Li , B. Handsaker , A. Wysoker , T. Fennell , J. Ruan , N. Homer , G. Marth , G. Abecasis , R. Durbin , Bioinformatics 2009, 25, 2078. 19505943 10.1093/bioinformatics/btp352 PMC2723002

[52] M. E. Skinner , A. V. Uzilov , L. D. Stein , C. J. Mungall , I. H. Holmes , Genome Res. 2009, 19, 1630. 19570905 10.1101/gr.094607.109 PMC2752129

