# Supplementary material for: Non‐Random Distribution of EMS‐Induced Mutations Reveals Preference for Open Chromatin and Expressed Genes in Rice
Source: Adv Sci (Weinh). 2025 Aug 13;12(39):e10034. doi: 10.1002/advs.202510034 (PMC12533142; doi:10.1002/advs.202510034)
Supplement: Supplementary file 1 — Supporting Information [file ADVS-12-e10034-s003.docx]

Supporting Information

for *Adv. Sci.*, DOI 10.1002/advs.202510034

Non-Random Distribution of EMS-Induced Mutations Reveals Preference for Open Chromatin and Expressed Genes in Rice

*Xue-Feng Yao*, Yanhong Liu, Zhiyong Li, Guo-Qiang Jiang, Wensheng Wang, Hong Lu, Huihui Li*, Zefu Lu* and Chun-Ming Liu**

**
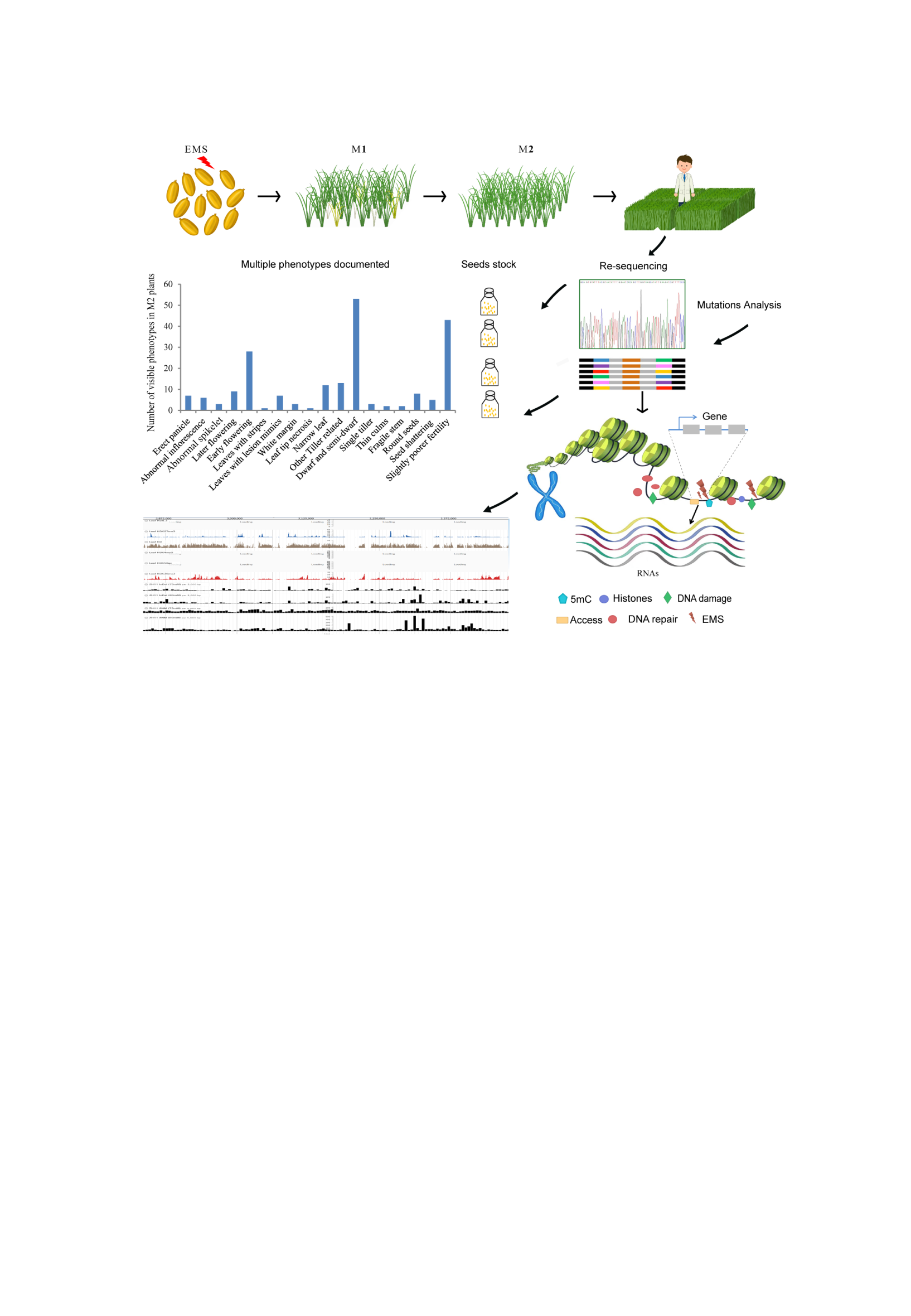
**

**Figure S1**. Overview of workflow in Rice4619.


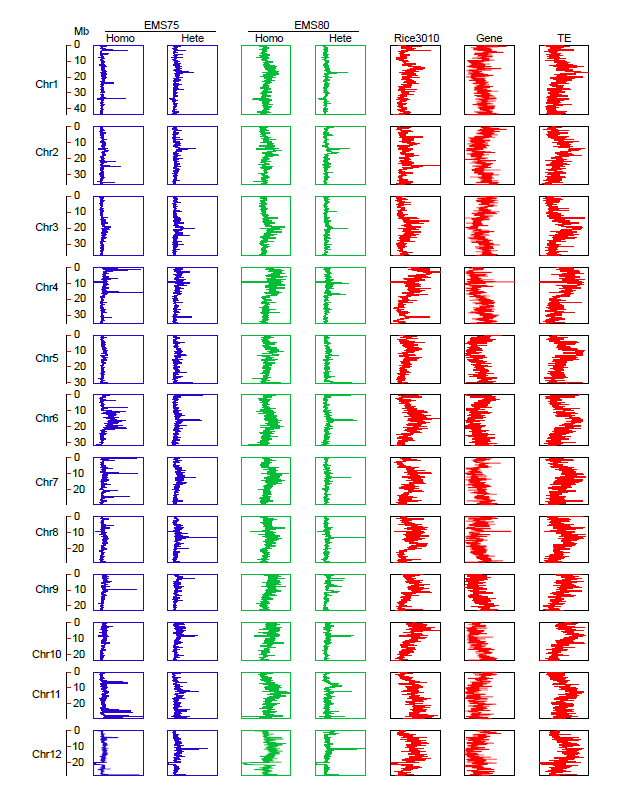


**Figure S2.** **Distributions of SNP^m^s in Rice4619**. Line plots showing distributions of SNP^m^s in Rice4619, SNPs in Rice3010, gene and repeated regions (TE) densities in the entire rice genome, with 100 Kb windows.


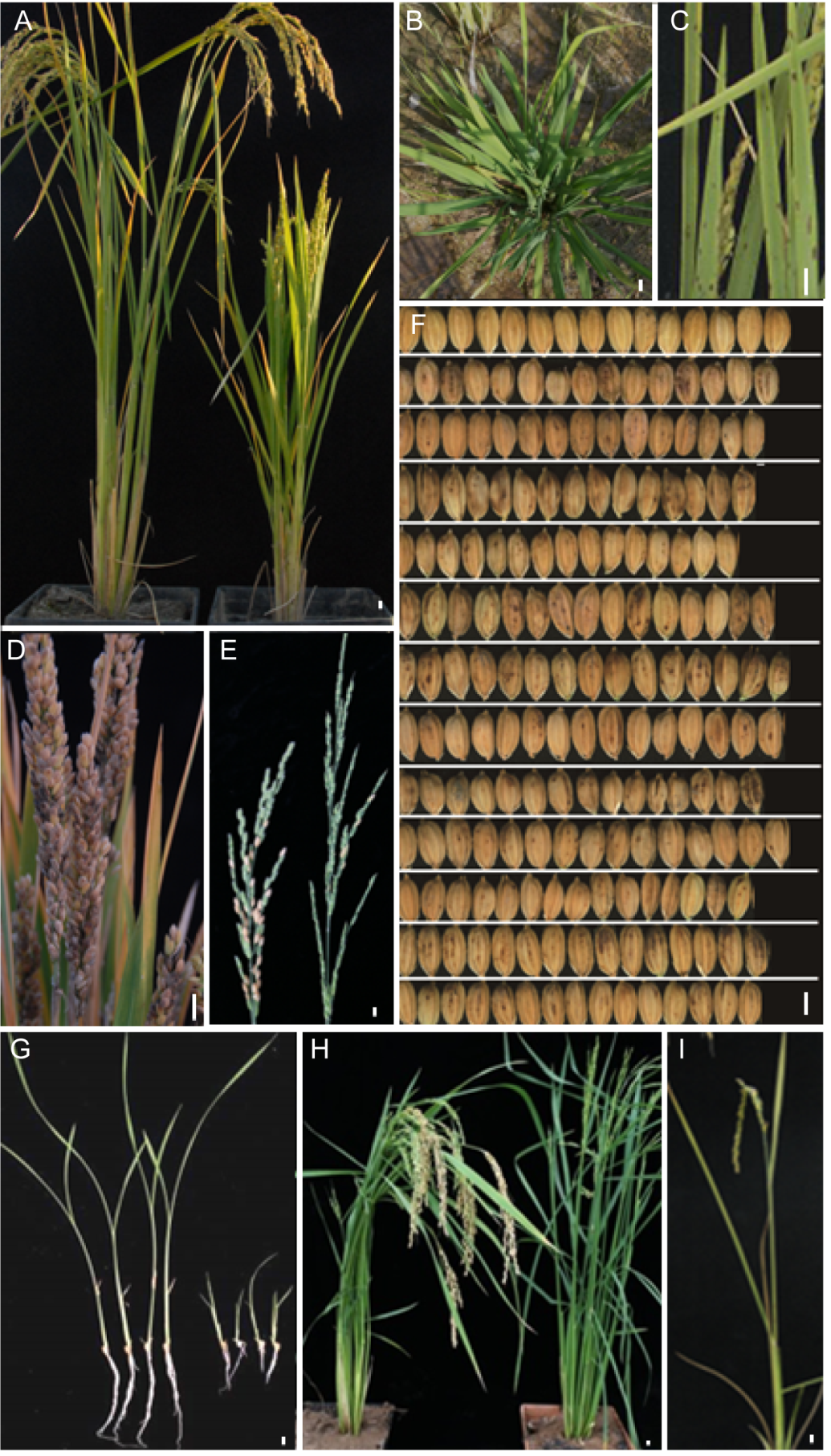


**Figure S3.** **Phenotypes observation in Rice4619**. A) to I), Basic phenotype documentations and various phenotypes of rice mutants derived from Rice4619 include variations in plant height (A), creeping growth (B), lesion mimics (C), dense panicle (D), panicle morphology and fertility (E), grain size (F), vigor of germination (G), flowering time (H), and tiller number (I). Scale bars, 1 cm.

**
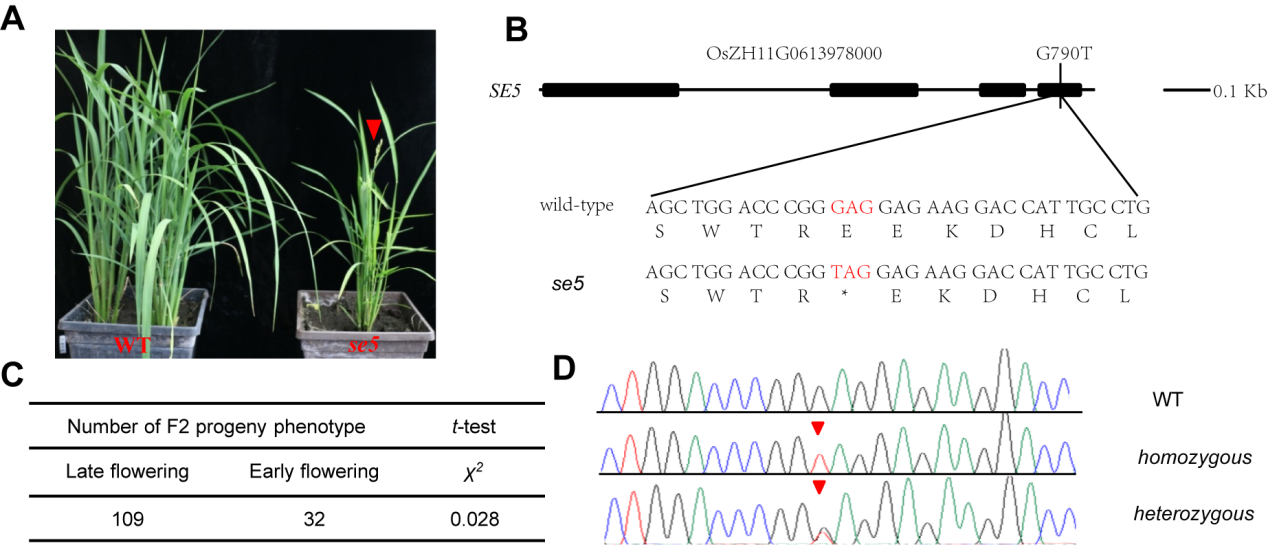
**

**Figure S4**. **Identification of heading date phenotype of *se5***. A) Phenotype of wild type (left) and early flowering mutant (red arrow head, right) were grown for 45 days after sowing under long-day conditions. B) The premature translational termination E264* was found in early flowering mutant line by Rice4619 sequencing data. C) The segregant analysis of early flowering phenotype (*P* < 0.05). D) Genotypes were confirmed by Sanger sequencing.


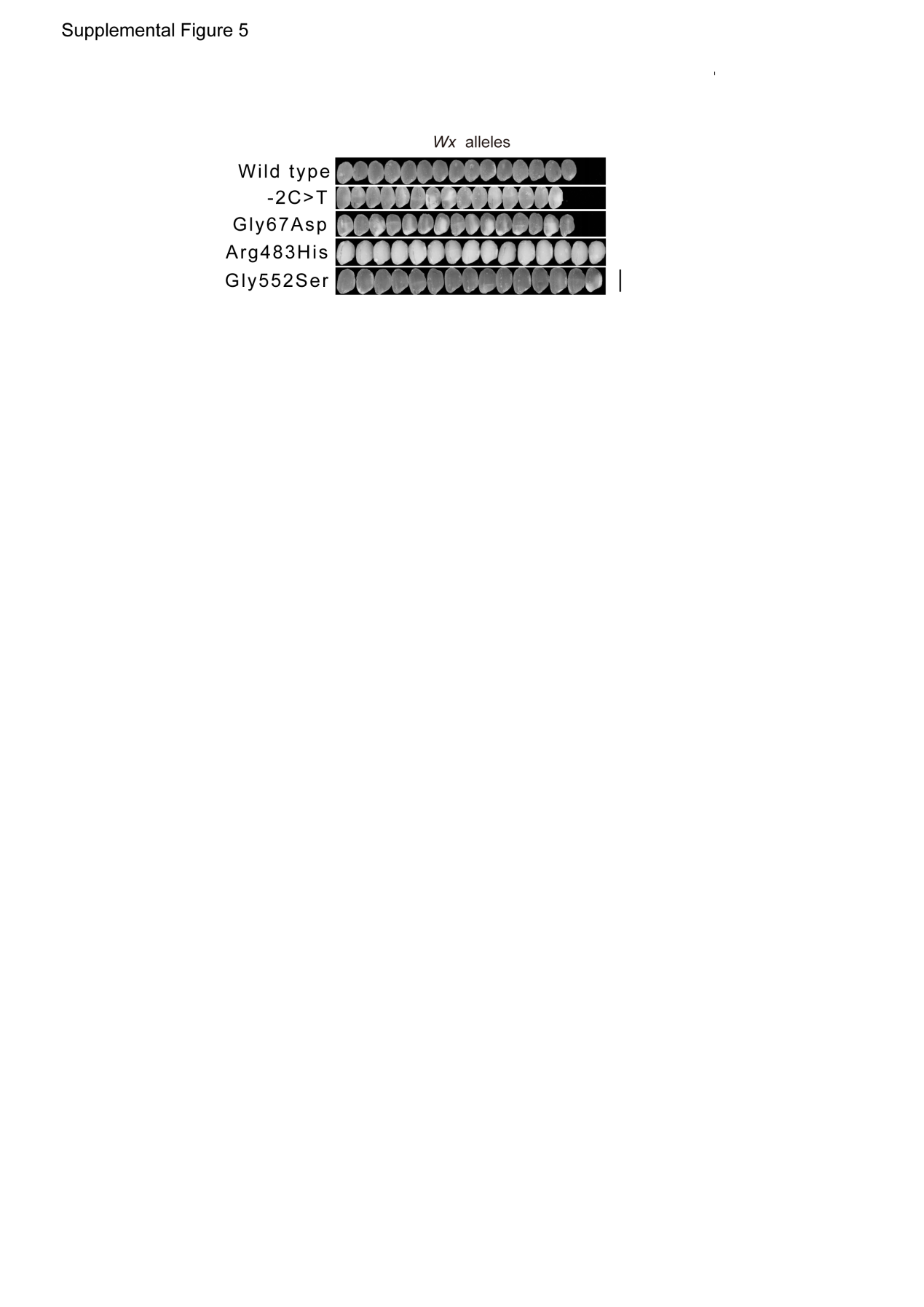


**Figure S5.** Identification of waxy phenotype in *Wx* alleles. Four alleles of *Wx* (*GBSSI*, *OsZH11G0611685100*) were identified in Rice4619, including a C/T substitution in 5’UTR; a Gly to Asp substitutions at the 67^th^ position; an Arg to His substitution at the 483^rd^ position; a Gly to Ser substitution at the 552^nd^ position. Arg483His mutations appear to have resulted in the loss-of-function waxy phenotype. All alleles were backcrossed to the wild-type of ZH11 to achieve a homozygous genotype. Scale bar, 0.5 cm.


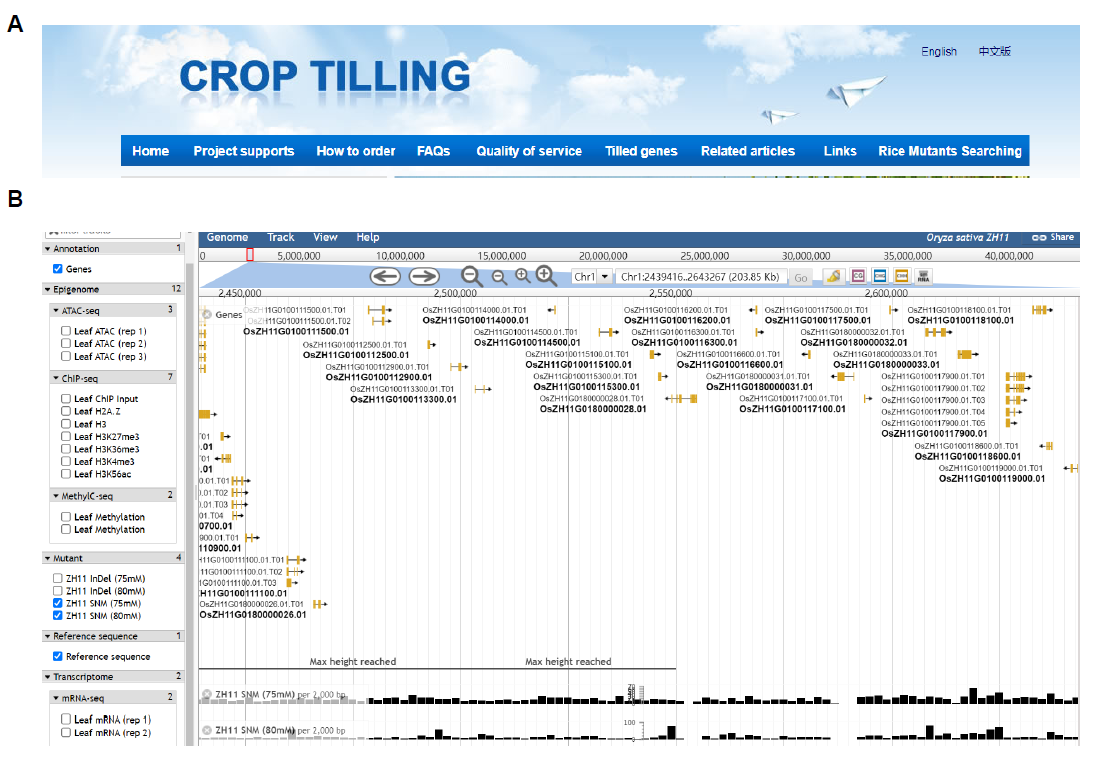


**Figure S6.** **Navigation tools in CROPTILLING database**. A) The main Navigation page of database of CROPTILLING. B) A JBrowser snapshot of mutations and epigenetic modifications in rice genome. Mutants can be searched using locus ID.


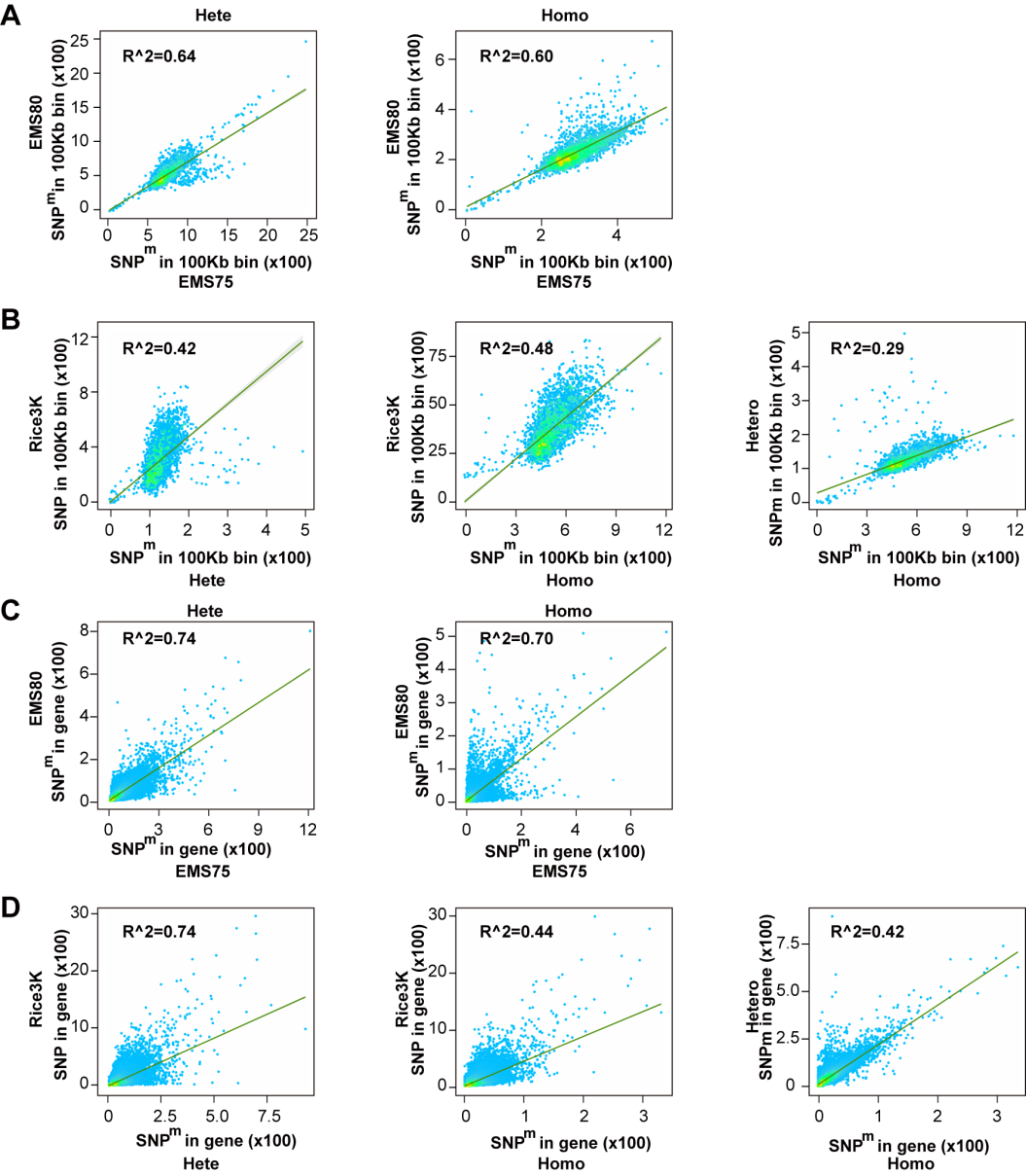


**Figure S7**. **Correlation analysis of SNP^m^ and SNP densities in Rice3010; heterozygous and homozygous mutations in Rice4619**. A) and B) show the correlations of variation densities within 100 Kb bins between EMS75 and EMS80 (A), and between SNP^m^s and SNPs in Rice3010 and Rice4619 (B). C) and D) display correlations in genebody regions, with SNP^m^s categorized by heterozygous and homozygous mutations.


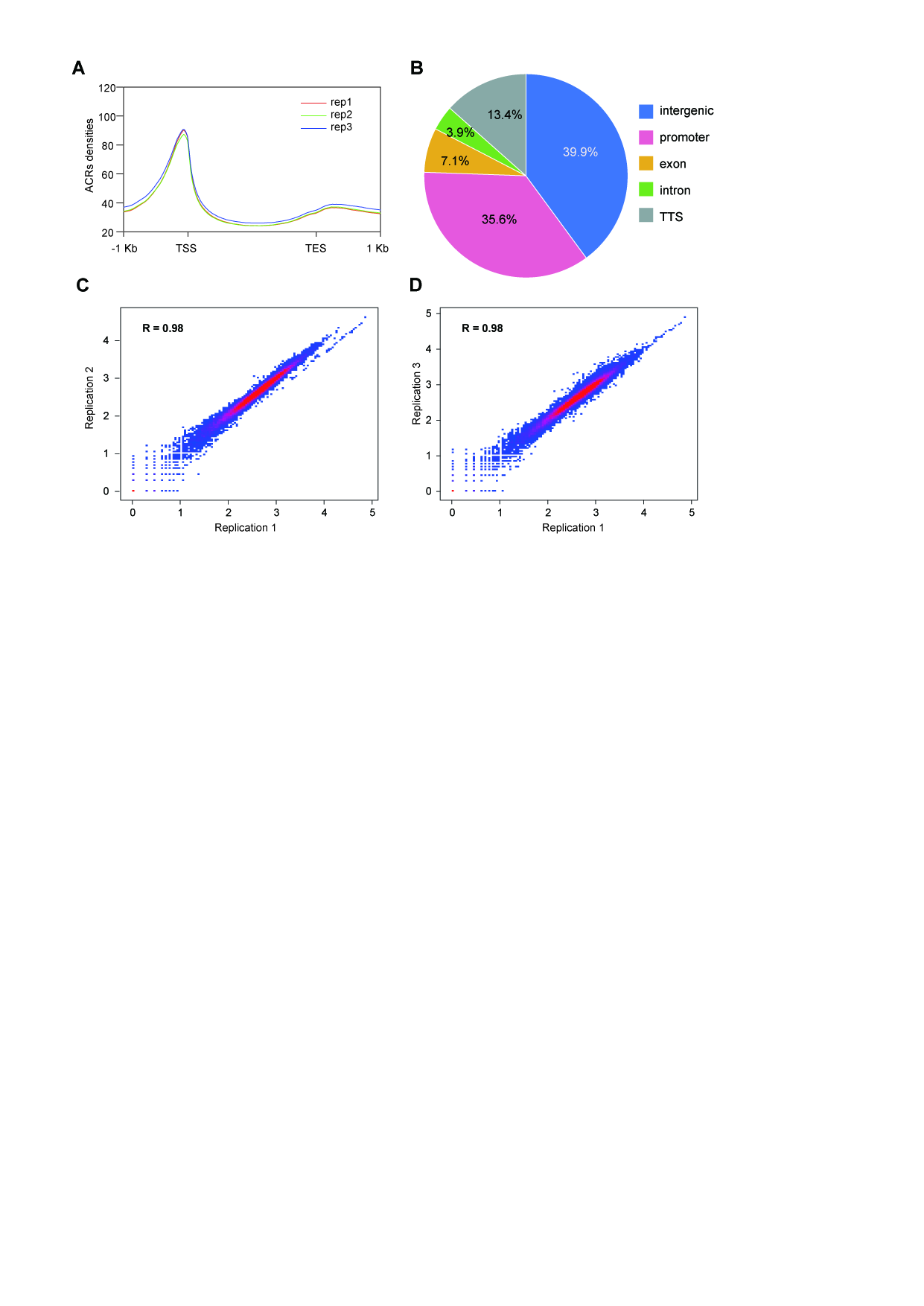


**Figure S8.** **Quality overview of ATAC-seq in ZH11**. The distribution of ACRs around genebodies detected by ATAC-seq in 7-day-old seedlings across three biological replicates. A) The X-axis represents the distance from the transcription start site (TSS), with -2.0 indicating 2 Kb upstream and +2.0 indicating 2 Kb downstream of the transcription termination site (TTS); the Y-axis shows the relative ATAC-seq signal values. B) A pie chart illustrating the distribution of ACRs across different genomic features, including intergenic regions, promoters, exons, introns, and TTS. C) and D) Correlation analysis of ATAC-seq data across the three biological replicates.


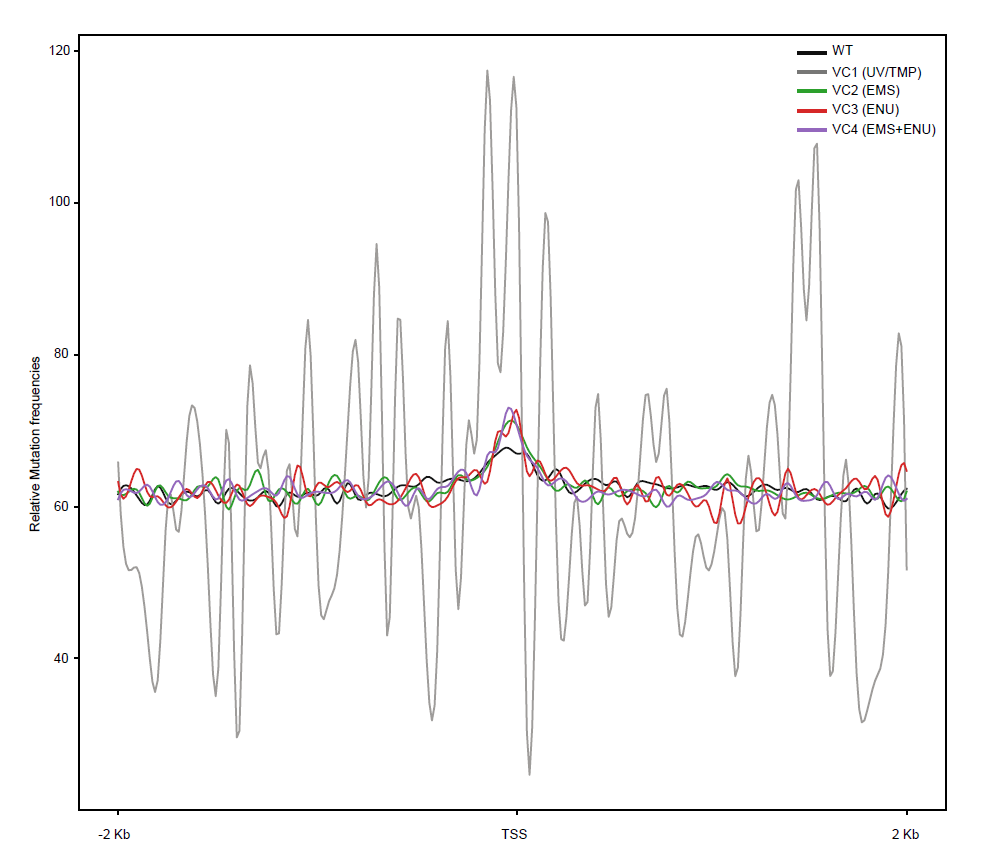


**Figure S9.** A metaplot showing induced mutation distributions around TSSs in *C. elegans* (wild isolates/WT, trimethylpsoralen/UV (TMP/UV), EMS, ENU and EMS+ENU). Note the elevated mutation frequencies in regions before the TSS in EMS, ENU and EMS+ENU populations, but not in WT and TMP/UV group. WT, wild isolates; VC1, TMP/UV; VC2, EMS; VC3, ENU; VC4, EMS+ENU. All mutations resource was derived from the Million Mutation Project, available at http://genome.sfu.ca/mmp. The average enrichment values around *C. elegans* genes in 4 Kb windows, and each of them was presented in 50-bp bins. The WS235 annotation gff3 file was available at https://ftp.ensemblgenomes.ebi.ac.uk/pub/metazoa/release-61/gff3/caenorhabditis_elegans/.
