# Supplementary material for: Non‐Random Distribution of EMS‐Induced Mutations Reveals Preference for Open Chromatin and Expressed Genes in Rice
Source: Adv Sci (Weinh). 2025 Aug 13;12(39):e10034. doi: 10.1002/advs.202510034 (PMC12533142; doi:10.1002/advs.202510034)
Supplement: Supplementary file 2 — Supplementary Table S1–S6 [file ADVS-12-e10034-s005.ppt]

## Slide 1
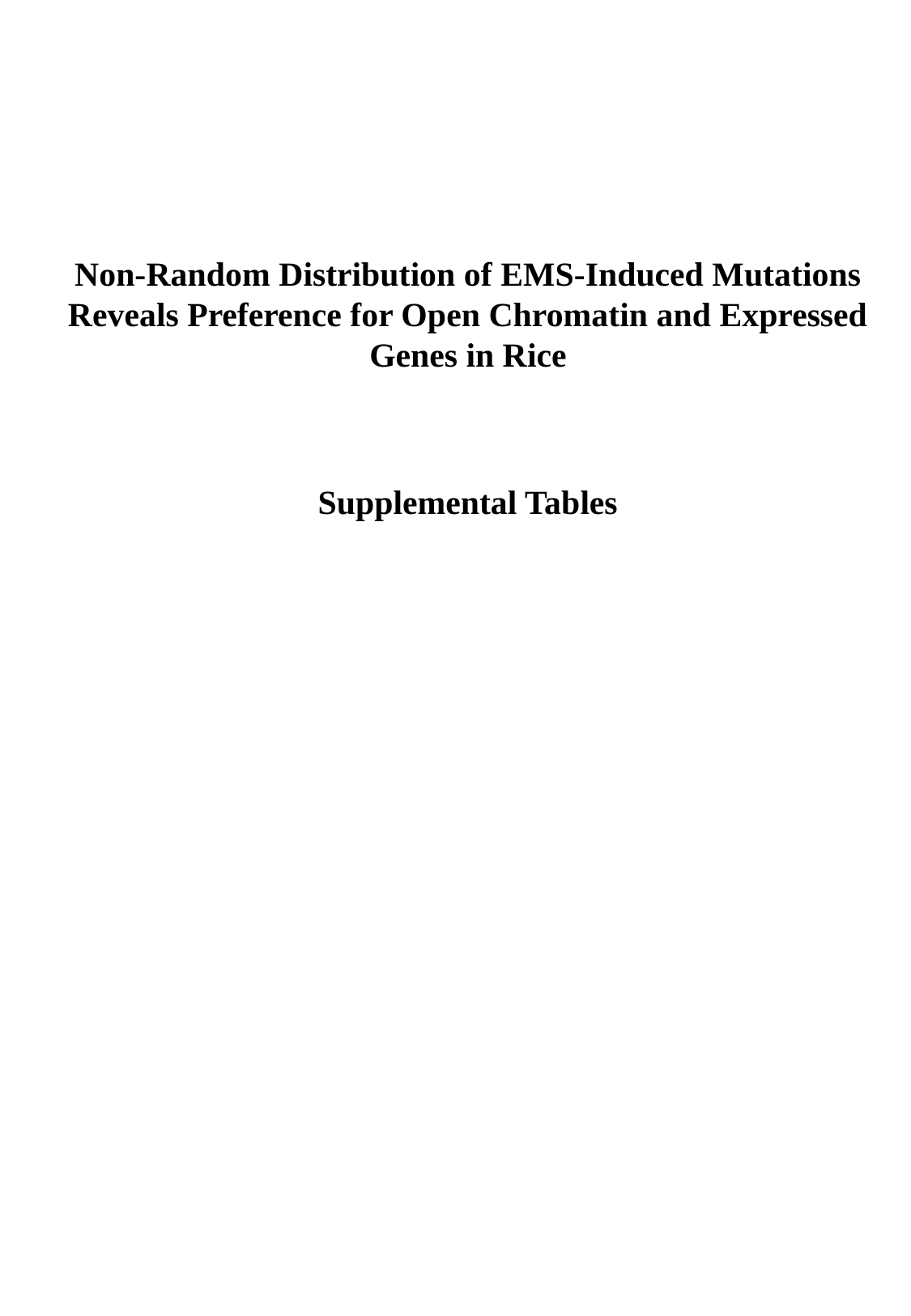

# Non-Random Distribution of EMS-Induced Mutations Reveals Preference for Open Chromatin and Expressed Genes in Rice
Supplemental Tables

## Slide 2
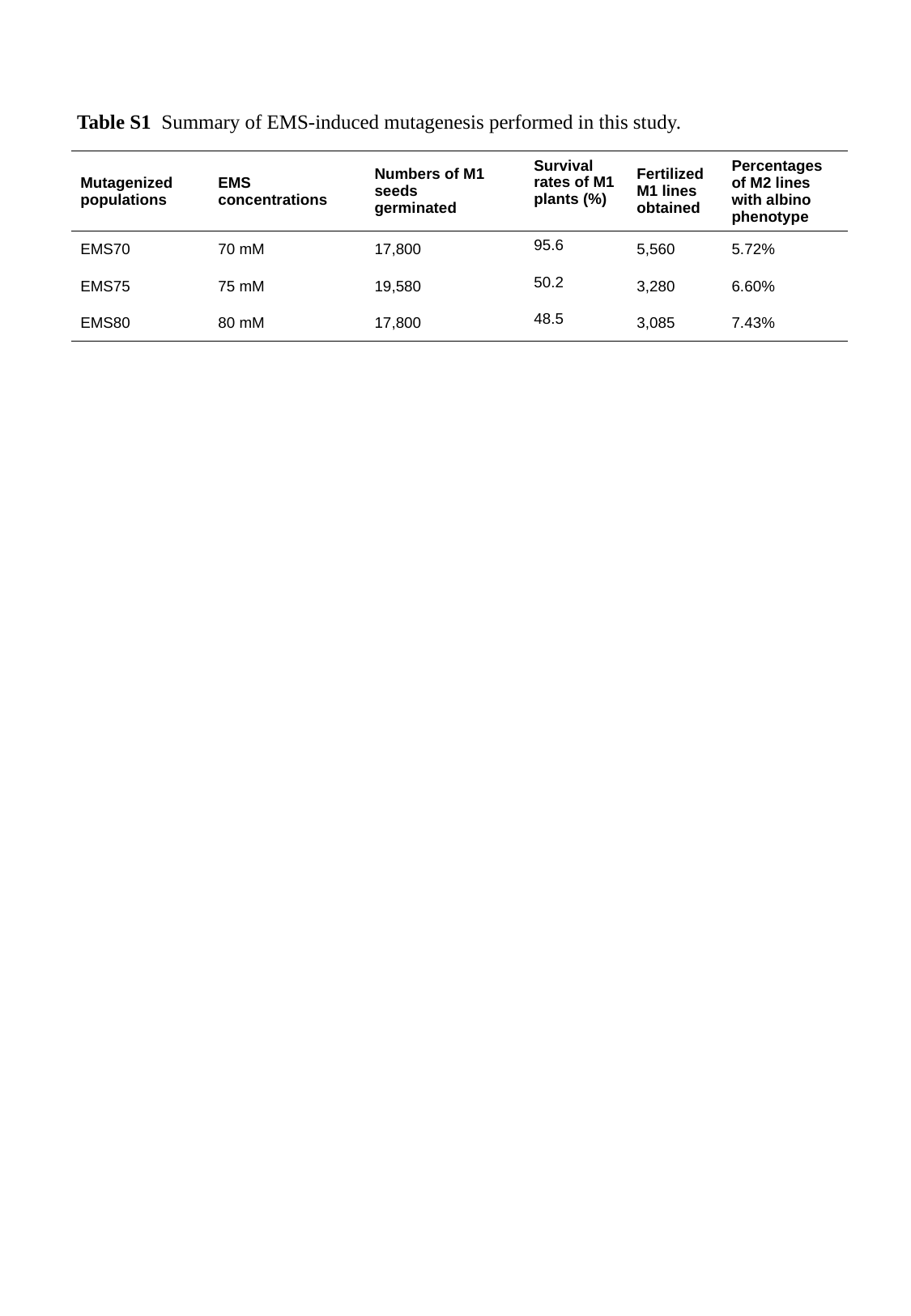

Table S1 Summary of EMS-induced mutagenesis performed in this study.
| Mutagenized populations | EMS concentrations | Numbers of M1 seeds germinated | Survival rates of M1 plants (%) | Fertilized M1 lines obtained | Percentages of M2 lines with albino phenotype |
| --- | --- | --- | --- | --- | --- |
| EMS70 | 70 mM | 17,800 | 95.6 | 5,560 | 5.72% |
| EMS75 | 75 mM | 19,580 | 50.2 | 3,280 | 6.60% |
| EMS80 | 80 mM | 17,800 | 48.5 | 3,085 | 7.43% |

## Slide 3
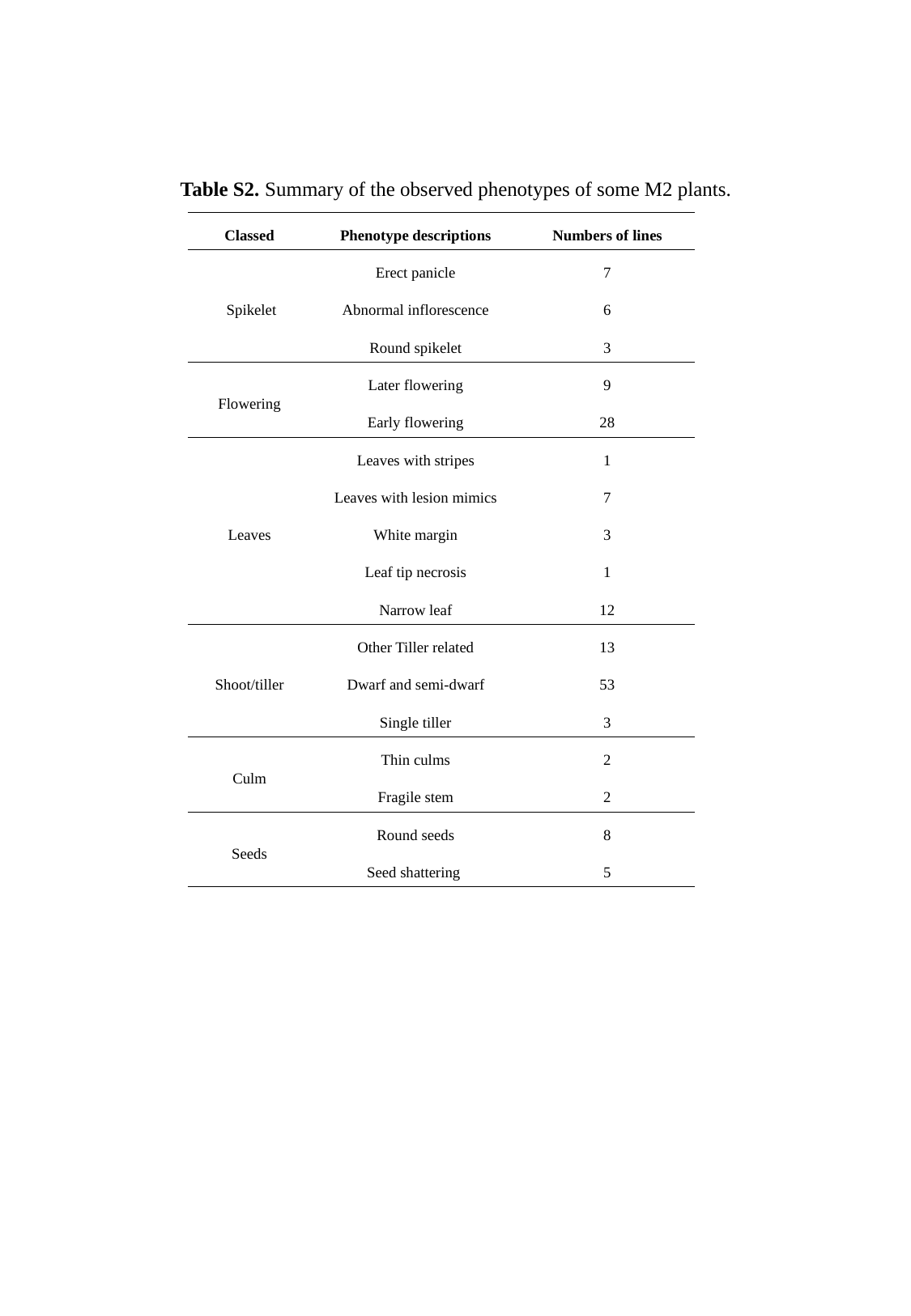

Table S2. Summary of the observed phenotypes of some M2 plants.
| Classed | Phenotype descriptions | Numbers of lines |
| --- | --- | --- |
| Spikelet | Erect panicle | 7 |
| | Abnormal inflorescence | 6 |
| | Round spikelet | 3 |
| Flowering | Later flowering | 9 |
| | Early flowering | 28 |
| Leaves | Leaves with stripes | 1 |
| | Leaves with lesion mimics | 7 |
| | White margin | 3 |
| | Leaf tip necrosis | 1 |
| | Narrow leaf | 12 |
| Shoot/tiller | Other Tiller related | 13 |
| | Dwarf and semi-dwarf | 53 |
| | Single tiller | 3 |
| Culm | Thin culms | 2 |
| | Fragile stem | 2 |
| Seeds | Round seeds | 8 |
| | Seed shattering | 5 |

## Slide 4
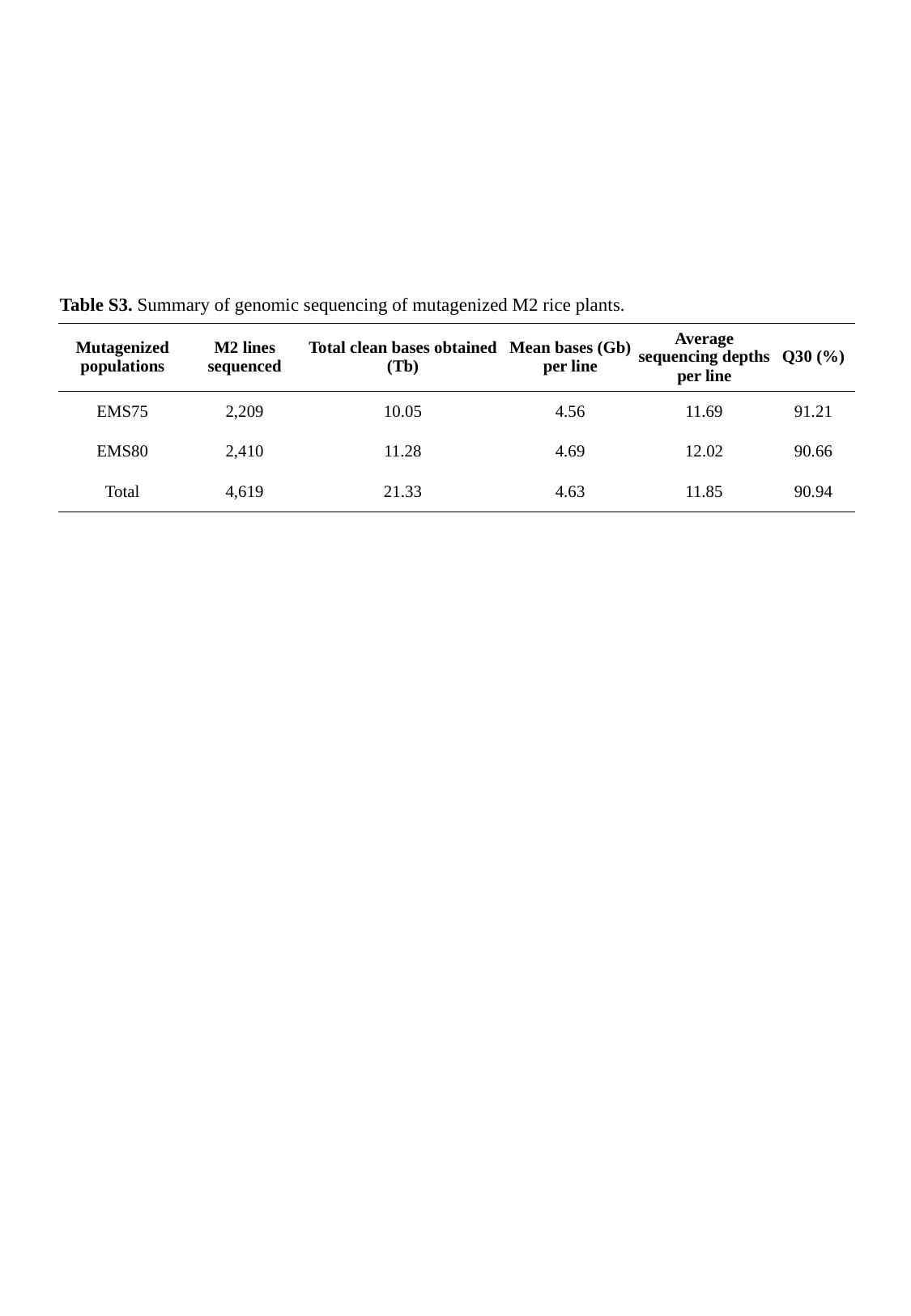

Table S3. Summary of genomic sequencing of mutagenized M2 rice plants.
| Mutagenized populations | M2 lines sequenced | Total clean bases obtained (Tb) | Mean bases (Gb) per line | Average sequencing depths per line | Q30 (%) |
| --- | --- | --- | --- | --- | --- |
| EMS75 | 2,209 | 10.05 | 4.56 | 11.69 | 91.21 |
| EMS80 | 2,410 | 11.28 | 4.69 | 12.02 | 90.66 |
| Total | 4,619 | 21.33 | 4.63 | 11.85 | 90.94 |

## Slide 5
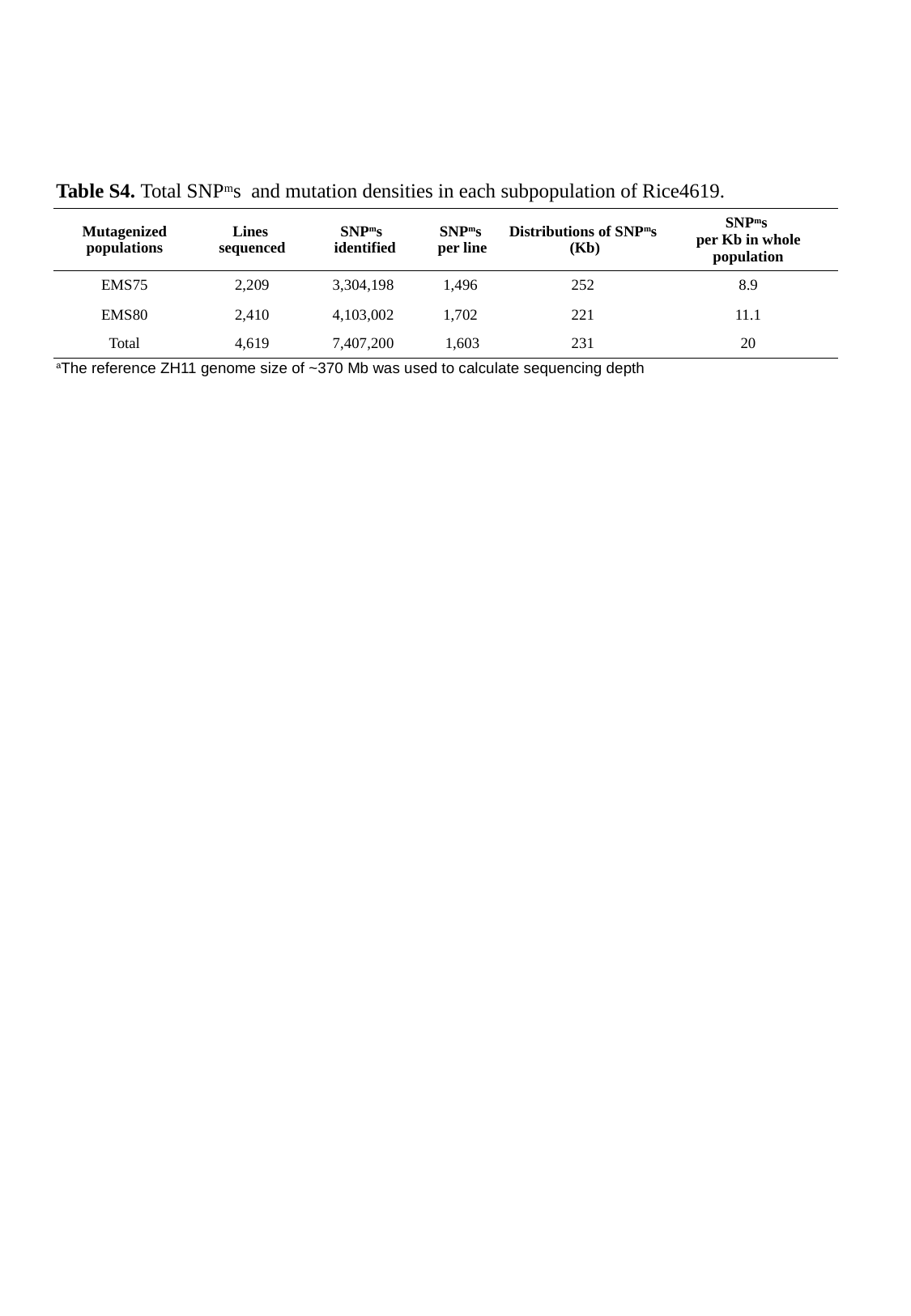

Table S4. Total SNPms and mutation densities in each subpopulation of Rice4619.
| Mutagenized populations | Lines sequenced | SNPms identified | SNPms per line | Distributions of SNPms (Kb) | SNPms per Kb in whole population |
| --- | --- | --- | --- | --- | --- |
| EMS75 | 2,209 | 3,304,198 | 1,496 | 252 | 8.9 |
| EMS80 | 2,410 | 4,103,002 | 1,702 | 221 | 11.1 |
| Total | 4,619 | 7,407,200 | 1,603 | 231 | 20 |
aThe reference ZH11 genome size of ~370 Mb was used to calculate sequencing depth

## Slide 6
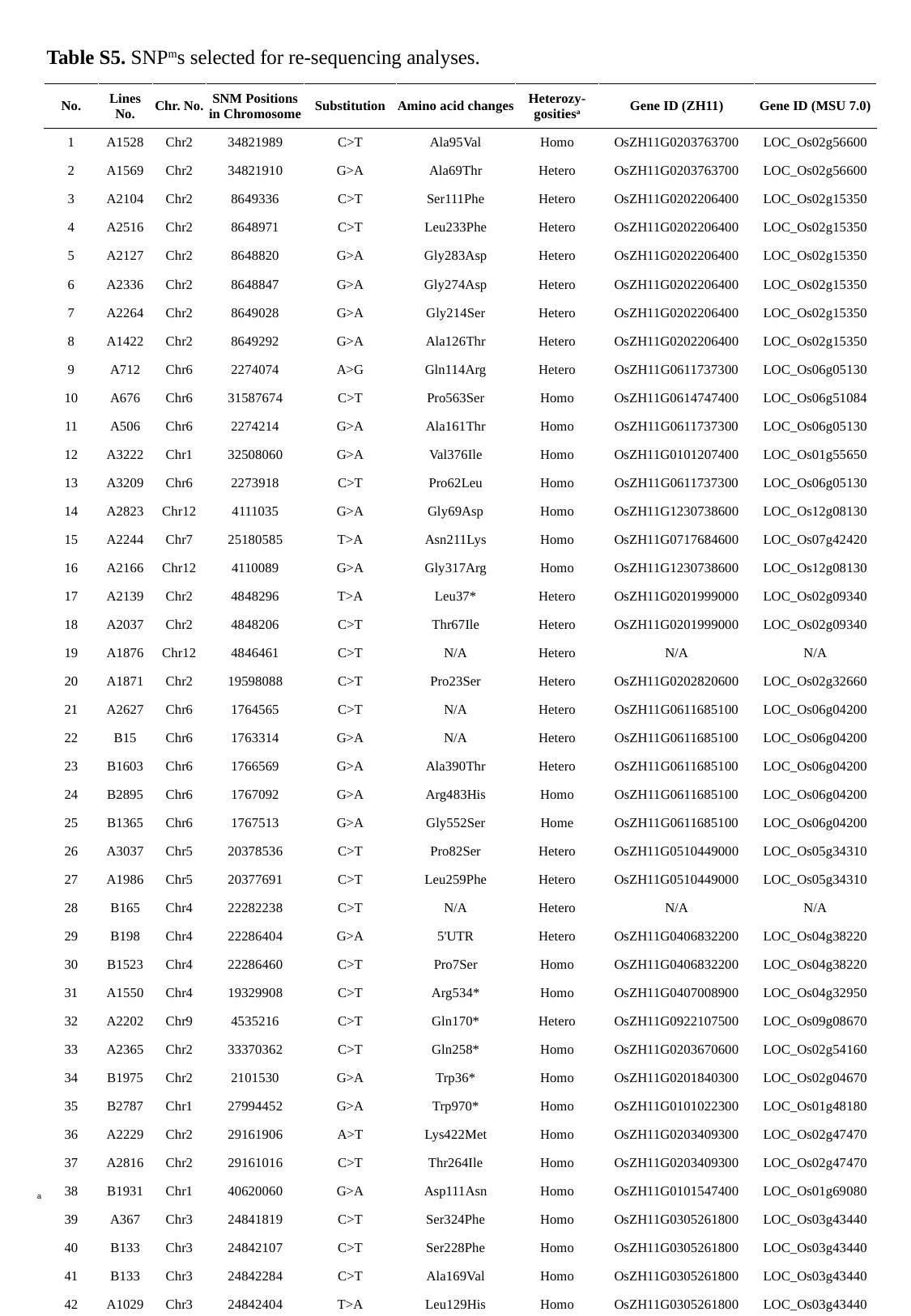

Table S5. SNPms selected for re-sequencing analyses.
| No. | Lines No. | Chr. No. | SNM Positions in Chromosome | Substitution | Amino acid changes | Heterozy-gositiesa | Gene ID (ZH11) | Gene ID (MSU 7.0) |
| --- | --- | --- | --- | --- | --- | --- | --- | --- |
| 1 | A1528 | Chr2 | 34821989 | C>T | Ala95Val | Homo | OsZH11G0203763700 | LOC\_Os02g56600 |
| 2 | A1569 | Chr2 | 34821910 | G>A | Ala69Thr | Hetero | OsZH11G0203763700 | LOC\_Os02g56600 |
| 3 | A2104 | Chr2 | 8649336 | C>T | Ser111Phe | Hetero | OsZH11G0202206400 | LOC\_Os02g15350 |
| 4 | A2516 | Chr2 | 8648971 | C>T | Leu233Phe | Hetero | OsZH11G0202206400 | LOC\_Os02g15350 |
| 5 | A2127 | Chr2 | 8648820 | G>A | Gly283Asp | Hetero | OsZH11G0202206400 | LOC\_Os02g15350 |
| 6 | A2336 | Chr2 | 8648847 | G>A | Gly274Asp | Hetero | OsZH11G0202206400 | LOC\_Os02g15350 |
| 7 | A2264 | Chr2 | 8649028 | G>A | Gly214Ser | Hetero | OsZH11G0202206400 | LOC\_Os02g15350 |
| 8 | A1422 | Chr2 | 8649292 | G>A | Ala126Thr | Hetero | OsZH11G0202206400 | LOC\_Os02g15350 |
| 9 | A712 | Chr6 | 2274074 | A>G | Gln114Arg | Hetero | OsZH11G0611737300 | LOC\_Os06g05130 |
| 10 | A676 | Chr6 | 31587674 | C>T | Pro563Ser | Homo | OsZH11G0614747400 | LOC\_Os06g51084 |
| 11 | A506 | Chr6 | 2274214 | G>A | Ala161Thr | Homo | OsZH11G0611737300 | LOC\_Os06g05130 |
| 12 | A3222 | Chr1 | 32508060 | G>A | Val376Ile | Homo | OsZH11G0101207400 | LOC\_Os01g55650 |
| 13 | A3209 | Chr6 | 2273918 | C>T | Pro62Leu | Homo | OsZH11G0611737300 | LOC\_Os06g05130 |
| 14 | A2823 | Chr12 | 4111035 | G>A | Gly69Asp | Homo | OsZH11G1230738600 | LOC\_Os12g08130 |
| 15 | A2244 | Chr7 | 25180585 | T>A | Asn211Lys | Homo | OsZH11G0717684600 | LOC\_Os07g42420 |
| 16 | A2166 | Chr12 | 4110089 | G>A | Gly317Arg | Homo | OsZH11G1230738600 | LOC\_Os12g08130 |
| 17 | A2139 | Chr2 | 4848296 | T>A | Leu37\* | Hetero | OsZH11G0201999000 | LOC\_Os02g09340 |
| 18 | A2037 | Chr2 | 4848206 | C>T | Thr67Ile | Hetero | OsZH11G0201999000 | LOC\_Os02g09340 |
| 19 | A1876 | Chr12 | 4846461 | C>T | N/A | Hetero | N/A | N/A |
| 20 | A1871 | Chr2 | 19598088 | C>T | Pro23Ser | Hetero | OsZH11G0202820600 | LOC\_Os02g32660 |
| 21 | A2627 | Chr6 | 1764565 | C>T | N/A | Hetero | OsZH11G0611685100 | LOC\_Os06g04200 |
| 22 | B15 | Chr6 | 1763314 | G>A | N/A | Hetero | OsZH11G0611685100 | LOC\_Os06g04200 |
| 23 | B1603 | Chr6 | 1766569 | G>A | Ala390Thr | Hetero | OsZH11G0611685100 | LOC\_Os06g04200 |
| 24 | B2895 | Chr6 | 1767092 | G>A | Arg483His | Homo | OsZH11G0611685100 | LOC\_Os06g04200 |
| 25 | B1365 | Chr6 | 1767513 | G>A | Gly552Ser | Home | OsZH11G0611685100 | LOC\_Os06g04200 |
| 26 | A3037 | Chr5 | 20378536 | C>T | Pro82Ser | Hetero | OsZH11G0510449000 | LOC\_Os05g34310 |
| 27 | A1986 | Chr5 | 20377691 | C>T | Leu259Phe | Hetero | OsZH11G0510449000 | LOC\_Os05g34310 |
| 28 | B165 | Chr4 | 22282238 | C>T | N/A | Hetero | N/A | N/A |
| 29 | B198 | Chr4 | 22286404 | G>A | 5'UTR | Hetero | OsZH11G0406832200 | LOC\_Os04g38220 |
| 30 | B1523 | Chr4 | 22286460 | C>T | Pro7Ser | Homo | OsZH11G0406832200 | LOC\_Os04g38220 |
| 31 | A1550 | Chr4 | 19329908 | C>T | Arg534\* | Homo | OsZH11G0407008900 | LOC\_Os04g32950 |
| 32 | A2202 | Chr9 | 4535216 | C>T | Gln170\* | Hetero | OsZH11G0922107500 | LOC\_Os09g08670 |
| 33 | A2365 | Chr2 | 33370362 | C>T | Gln258\* | Homo | OsZH11G0203670600 | LOC\_Os02g54160 |
| 34 | B1975 | Chr2 | 2101530 | G>A | Trp36\* | Homo | OsZH11G0201840300 | LOC\_Os02g04670 |
| 35 | B2787 | Chr1 | 27994452 | G>A | Trp970\* | Homo | OsZH11G0101022300 | LOC\_Os01g48180 |
| 36 | A2229 | Chr2 | 29161906 | A>T | Lys422Met | Homo | OsZH11G0203409300 | LOC\_Os02g47470 |
| 37 | A2816 | Chr2 | 29161016 | C>T | Thr264Ile | Homo | OsZH11G0203409300 | LOC\_Os02g47470 |
| 38 | B1931 | Chr1 | 40620060 | G>A | Asp111Asn | Homo | OsZH11G0101547400 | LOC\_Os01g69080 |
| 39 | A367 | Chr3 | 24841819 | C>T | Ser324Phe | Homo | OsZH11G0305261800 | LOC\_Os03g43440 |
| 40 | B133 | Chr3 | 24842107 | C>T | Ser228Phe | Homo | OsZH11G0305261800 | LOC\_Os03g43440 |
| 41 | B133 | Chr3 | 24842284 | C>T | Ala169Val | Homo | OsZH11G0305261800 | LOC\_Os03g43440 |
| 42 | A1029 | Chr3 | 24842404 | T>A | Leu129His | Homo | OsZH11G0305261800 | LOC\_Os03g43440 |
| 43 | B23 | Chr3 | 24842413 | G>A | Arg126Gln | Hetero | OsZH11G0305261800 | LOC\_Os03g43440 |
| 44 | A2549 | Chr6 | 1608221 | G>A | Lys700Lys | Homo | OsZH11G0611665900 | LOC\_Os06g03970 |
| 45 | A27 | Chr6 | 1608481 | C>T | Pro760Ser | Homo | OsZH11G0611665900 | LOC\_Os06g03970 |
| 46 | B1572 | Chr6 | 1608627 | C>T | Asn808Asn | Homo | OsZH11G0611665900 | LOC\_Os06g03970 |
| 47 | A2344 | Chr6 | 1608994 | C>T | Ala887Val | Homo | OsZH11G0611665900 | LOC\_Os06g03970 |
| 48 | B2276 | Chr6 | 1609062 | G>A | Val910Ile | Homo | OsZH11G0611665900 | LOC\_Os06g03970 |
| 49 | B974 | Chr1 | 26228930 | C>T | Pro153Leu | Hetero | OsZH11G0100963600 | LOC\_Os01g45530 |
| 50 | A1293 | Chr1 | 36827229 | C>T | Ala227Val | Homo | OsZH11G0101395300 | LOC\_Os01g62780 |
| 51 | A2831 | Chr1 | 36827526 | G>A | Ser326Asn | Homo | OsZH11G0101395300 | LOC\_Os01g62780 |
| 52 | B324 | Chr1 | 36827232 | C>T | Ala228Val | Homo | OsZH11G0101395300 | LOC\_Os01g62780 |
| 53 | B1271 | Chr1 | 36827183 | G>A | Ala212Thr | Hetero | OsZH11G0101395300 | LOC\_Os01g62780 |
| 54 | A1211 | Chr2 | 31235801 | G>A | Ser485Asn | Homo | OsZH11G0203543700 | LOC\_Os02g50840 |
| 55 | A3056 | Chr2 | 31236299 | C>T | Thr651Ile | Homo | OsZH11G0203543700 | LOC\_Os02g50840 |
a Homo, homozygous; Hetero, heterozygous

## Slide 7
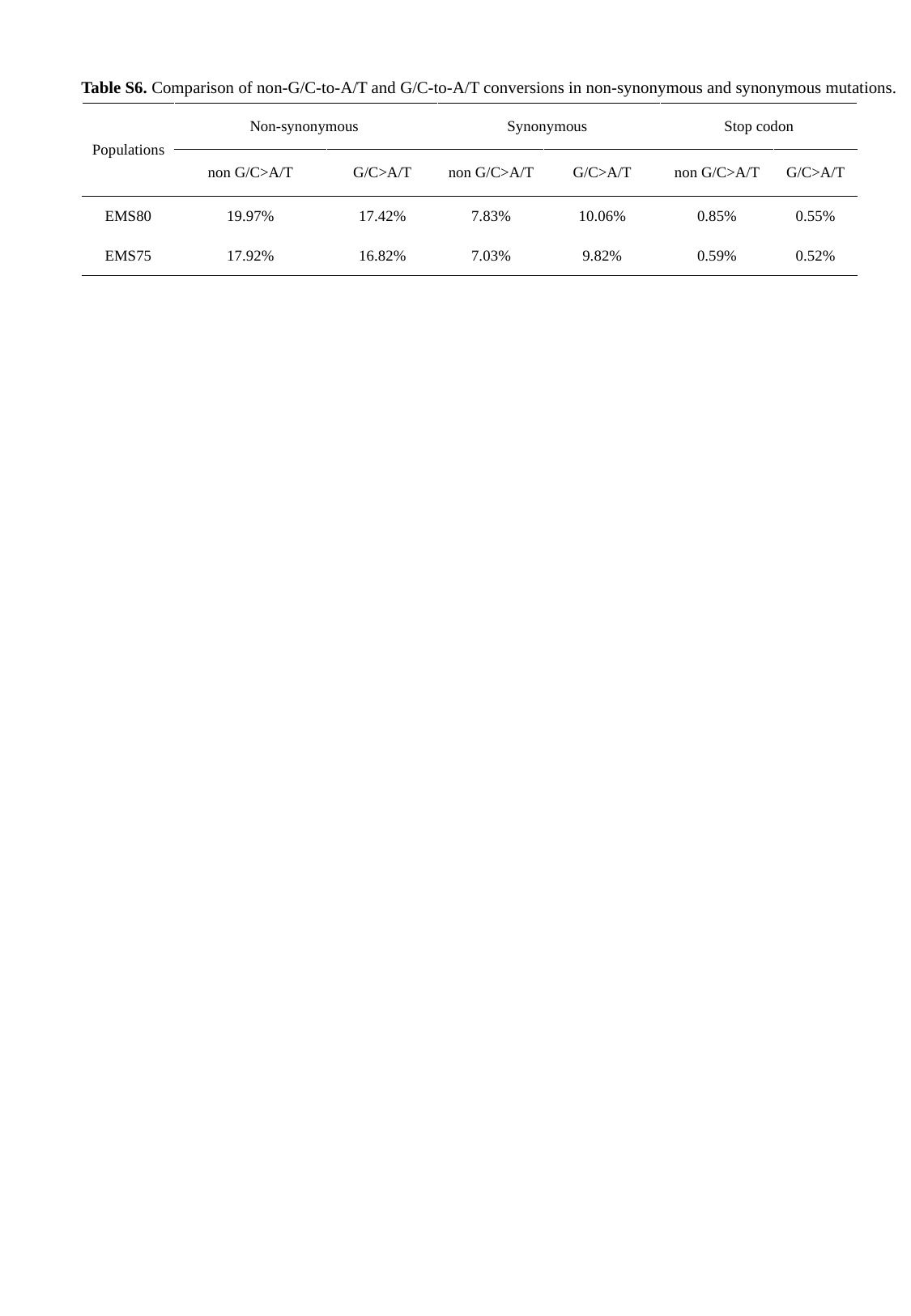

Table S6. Comparison of non-G/C-to-A/T and G/C-to-A/T conversions in non-synonymous and synonymous mutations.
| Populations | Non-synonymous | | Synonymous | | Stop codon | |
| --- | --- | --- | --- | --- | --- | --- |
| | non G/C>A/T | G/C>A/T | non G/C>A/T | G/C>A/T | non G/C>A/T | G/C>A/T |
| EMS80 | 19.97% | 17.42% | 7.83% | 10.06% | 0.85% | 0.55% |
| EMS75 | 17.92% | 16.82% | 7.03% | 9.82% | 0.59% | 0.52% |
